# Human BioMolecular Atlas Program (HuBMAP): 3D Human Reference Atlas construction and usage

**DOI:** 10.1038/s41592-024-02563-5

**Published:** 2025-03-13

**Authors:** Katy Börner, Philip D. Blood, Jonathan C. Silverstein, Matthew Ruffalo, Rahul Satija, Sarah A. Teichmann, Gloria J. Pryhuber, Ravi S. Misra, Jeffrey M. Purkerson, Jean Fan, John W. Hickey, Gesmira Molla, Chuan Xu, Yun Zhang, Griffin M. Weber, Yashvardhan Jain, Danial Qaurooni, Yongxin Kong, HRA Team, Jakub Abramson, David Anderson, Kristin Ardlie, Mark J. Arends, Bruce J. Aronow, Rachel Bajema, Richard A. Baldock, Ross Barnowski, Daria Barwinska, Amy Bernard, David Betancur, Supriya Bidanta, Frida Björklund, Axel Bolin, Avinash Boppana, Luke Boulter, Kristen Browne, Maigan A. Brusko, Albert Burger, Martha Campbell-Thompson, Ivan Cao-Berg, Anita R. Caron, Megan Carroll, Chrystal Chadwick, Haoran Chen, Lu Chen, Bernard de Bono, Gail Deutsch, Song-Lin Ding, Sean Donahue, Tarek M. El-Achkar, Adel Eskaros, Louis Falo, Melissa Farrow, Michael J. Ferkowicz, Stephen A. Fisher, James C. Gee, Ronald N. Germain, Michael Ginda, Fiona Ginty, Sarah A. Gitomer, Melanie B. Goldstone, Katherine S. Gustilo, James S. Hagood, Marc K. Halushka, Muzlifah A. Haniffa, Peter Hanna, Josef Hardi, Yongqun Oliver He, Brendan John Honick, Derek Houghton, Maxim Itkin, Sanjay Jain, Laura Jardine, Z. Gordon Jiang, Yingnan Ju, Arivarasan Karunamurthy, Neil L. Kelleher, Timothy J. Kendall, Angela R. S. Kruse, Monica M. Laronda, Louise C. Laurent, Elisa Laurenti, Sujin Lee, Ed Lein, Chenran Li, Zhuoyan Li, Shin Lin, Yiing Lin, Scott A. Lindsay, Teri A. Longacre, Emma Lundberg, Libby Maier, Rajeev Malhotra, Anna Martinez Casals, Anna Maria Masci, Clayton E. Mathews, Elizabeth McDonough, James A. McLaughlin, Rajasree Menon, Vilas Menon, Jeremy A. Miller, Richard Morgan, Werner Müller, Robert F. Murphy, Mark A. Musen, Harikrishna Nakshatri, Martijn C. Nawijn, Elizabeth K. Neumann, Debra J. Nigra, Kathleen O’Neill, Mana M. Parast, Ushma Patel, Liming Pei, Hemali Phatnani, Gesina A. Phillips, Alison M. Pouch, Alvin C. Powers, Juan F. Puerto, Aleix Puig-Barbe, Ellen M. Quardokus, Andrea J. Radtke, Presha Rajbhandari, Elizabeth G. Record, Drucilla J. Roberts, Alexander J. Ropelewski, David Rowe, Nancy L. Ruschman, Diane C. Saunders, Richard H. Scheuermann, Kevin L. Schey, Birgit Schilling, Heidi Schlehlein, Melissa Schwenk, Robin Scibek, Robert P. Seifert, Bill Shirey, Kalyanam Shivkumar, Kimberly Siletti, J. Alan Simmons, Dhruv Singhal, Michael Snyder, Jeffrey M. Spraggins, Valentina Stanley, Douglas W. Strand, Joel C. Sunshine, Christine Surrette, Ayako Suzuki, Purushothama Rao Tata, Deanne M. Taylor, Todd Theriault, Tracey Theriault, Jerin Easo Thomas, Elizabeth L. Tsui, Jackie Uranic, M. Todd Valerius, David Van Valen, Chad M. Vezina, Ioannis S. Vlachos, Fusheng Wang, Xuefei ‘Julie’ Wang, Clive H. Wasserfall, Joel S. Welling, Christopher Werlein, Seth Winfree, Devin M. Wright, Li Yao, Zhou Yuan, Ted Zhang, Andreas Bueckle, Bruce W. Herr

**Affiliations:** 1 Department of Intelligent Systems Engineering, Luddy School of Informatics, Computing, and Engineering, Indiana University, Bloomington, IN USA; 2 CIFAR MacMillan Multiscale Human program, CIFAR, Toronto, Ontario Canada; 3 Pittsburgh Supercomputing Center, Carnegie Mellon University, Pittsburgh, PA USA; 4 Department of Biomedical Informatics, University of Pittsburgh School of Medicine, Pittsburgh, PA USA; 5 Ray and Stephanie Lane Computational Biology Department, Carnegie Mellon University, Pittsburgh, PA USA; 6 New York Genome Center, New York, NY USA; 7 Cambridge Stem Cell Institute, Jeffrey Cheah Biomedical Centre, Cambridge Biomedical Campus, University of Cambridge, Cambridge, UK; 8 Department of Medicine, University of Cambridge, Cambridge, UK; 9 University of Rochester Medical Center, Rochester, NY USA; 10 Department of Biomedical Engineering, Johns Hopkins University, Baltimore, MD USA; 11 Department of Biomedical Engineering, Duke University, Durham, NC USA; 12 J. Craig Venter Institute, La Jolla, CA USA; 13 Department of Biomedical Informatics, Harvard Medical School, Boston, MA USA; 14 Weizmann Institute of Science, Rehovot, Israel; 15 Vanderbilt University, Nashville, TN USA; 16 Broad Institute, Cambridge, MA USA; 17 University of Edinburgh, Edinburgh, UK; 18 Cincinnati Children’s Hospital Medical Center, Cincinnati, OH USA; 19 Indiana University, Bloomington, IN USA; 20 California Institute of Technology, Pasadena, CA USA; 21 Indiana University School of Medicine, Indianapolis, IN USA; 22 The Kavli Foundation, Los Angeles, CA USA; 23 Stanford University, Stanford, CA USA; 24 National Institute of Allergy and Infectious Diseases, National Institutes of Health, Buffalo, NY USA; 25 University of Florida, Gainesville, FL USA; 26 Heriot-Watt University, Edinburgh, UK; 27 EMBL-EBI, Hinxton, Cambridgeshire, UK; 28 GE HealthCare Technology & Innovation Center, Niskayuna, NY USA; 29 Carnegie Mellon University, Pittsburgh, PA USA; 30 Stony Brook University, Stony Brook, NY USA; 31 University of Washington, Seattle, WA USA; 32 Allen Institute for Brain Science, Seattle, WA USA; 33 Indiana University School of Medicine & Indianapolis VA Medical Center, Indianapolis, IN USA; 34 Vanderbilt University Medical Center, Nashville, TN USA; 35 University of Pittsburgh, Pittsburgh, PA USA; 36 Vanderbilt University School of Medicine, Nashville, TN USA; 37 University of Pennsylvania, Philadelphia, PA USA; 38 National Institute of Allergy and Infectious Diseases, National Institutes of Health, Bethesda, MD USA; 39 University of Colorado School of Medicine, Aurora, CO USA; 40 University of North Carolina-Chapel Hill, Chapel Hill, NC USA; 41 Cleveland Clinic Foundation, Cleveland, OH USA; 42 Wellcome Sanger Institute, Cambridge, UK; 43 University of California, Los Angeles, Los Angeles, CA USA; 44 University of Michigan, Ann Arbor, MI USA; 45 Pittsburgh Supercomputing Center & Carnegie Mellon University, Pittsburgh, PA USA; 46 Penn Medicine, University of Pennsylvania, Philadelphia, PA USA; 47 Washington University in St. Louis, St. Louis, MO USA; 48 Newcastle University, Newcastle upon Tyne, UK; 49 Beth Israel Deaconess Medical Center & Harvard Medical School, Boston, MA USA; 50 Northwestern University, Evanston, IL USA; 51 Ann and Robert H. Lurie Children’s Hospital of Chicago, Northwestern University, Chicago, IL USA; 52 University of California, San Diego, San Diego, CA USA; 53 University of Cambridge, Cambridge, UK; 54 Massachusetts General Hospital & Harvard Medical School, Boston, MA USA; 55 Allen Institute, Seattle, WA USA; 56 University of Texas MD Anderson Cancer Center, Houston, TX USA; 57 EMBL-EBI, Cambridge, UK; 58 University of Michigan Medical School, Ann Arbor, MI USA; 59 Columbia University Irving Medical Center, New York, NY USA; 60 University of Manchester, UK and Miltenyi Biotec, Germany, Bergisch-Gladbach, Germany; 61 University of Groningen, University Medical Center Groningen, Groningen, The Netherlands; 62 UC Davis, Davis, CA USA; 63 University of California San Diego, La Jolla, CA USA; 64 Children’s Hospital of Philadelphia, University of Pennsylvania, Philadelphia, PA USA; 65 Columbia University & New York Genome Center, New York City, NY USA; 66 Vanderbilt University, Vanderbilt University Medical Center, VA Tennessee Valley Healthcare System, Nashville, TN USA; 67 Leica Microsystems Inc., Wetzlar, Germany; 68 Columbia University, New York, NY USA; 69 Harvard Medical School-Mass General Brigham, Boston, MA USA; 70 University of Connecticut Health, Farmington, CT USA; 71 National Library of Medicine, National Institutes of Health, Bethesda, MD USA; 72 Buck Institute for Research on Aging, Novato, CA USA; 73 University of Florida College of Medicine, Gainesville, FL USA; 74 University Medical Center Utrecht, Utrecht, The Netherlands; 75 Harvard-Beth Israel Deaconess Medical Center, Boston, MA USA; 76 University of Texas Southwestern Medical Center, Dallas, TX USA; 77 Johns Hopkins University School of Medicine, Baltimore, MD USA; 78 Duke University School of Medicine, Durham, NC USA; 79 The Children’s Hospital of Philadelphia & University of Pennsylvania Perelman Medical School, Philadelphia, PA USA; 80 Northwestern University, Chicago, IL USA; 81 Brigham and Women’s Hospital, Harvard Medical School, Boston, MA USA; 82 University of Wisconsin-Madison, Madison, WI USA; 83 Hannover Medical School, Hannover, Germany; 84 National Institute of Allergy and Infectious Disease, National Institutes of Health, Rockville, MD USA; 85 Liyaovisuals Design Studio, Dewood, MD USA

**Keywords:** Data integration, Computational platforms and environments, Biochemistry, Cell biology, Physiology

## Abstract

The Human BioMolecular Atlas Program (HuBMAP) aims to construct a 3D Human Reference Atlas (HRA) of the healthy adult body. Experts from 20+ consortia collaborate to develop a Common Coordinate Framework (CCF), knowledge graphs and tools that describe the multiscale structure of the human body (from organs and tissues down to cells, genes and biomarkers) and to use the HRA to characterize changes that occur with aging, disease and other perturbations. HRA v.2.0 covers 4,499 unique anatomical structures, 1,195 cell types and 2,089 biomarkers (such as genes, proteins and lipids) from 33 ASCT+B tables and 65 3D Reference Objects linked to ontologies. New experimental data can be mapped into the HRA using (1) cell type annotation tools (for example, Azimuth), (2) validated antibody panels or (3) by registering tissue data spatially. This paper describes HRA user stories, terminology, data formats, ontology validation, unified analysis workflows, user interfaces, instructional materials, application programming interfaces, flexible hybrid cloud infrastructure and previews atlas usage applications.

## Abstract (web summary)

This Resource describes data, code and tools developed for the Human Reference Atlas and the Human BioMolecular Atlas Program for building and navigating a multiscale human atlas.

## Main

Inaugurated in 2018, the Human BioMolecular Atlas Program (HuBMAP) aims to construct a comprehensive reference model of the healthy (‘non-diseased’) human body across all levels, from organs and tissues down to cells and canonical biomarkers^1,2^. The HuBMAP Portal (https://hubmapconsortium.org) introduces goals and links to experimental and atlas data, tools and training materials. The Data Portal (https://portal.hubmapconsortium.org) serves experimental datasets and supports data processing, search, filtering and visualization. The Human Reference Atlas Portal (https://humanatlas.io) provides open access to atlas data, code, procedures and instructional materials. The Human Reference Atlas (HRA)^3^ includes a Common Coordinate Framework (CCF; see Box 1), which helps harmonize multimodal data, including three-dimensional (3D) organ models, histology images and omics data from profiling of single cells. HRA data comprise human-expert-generated information (for example, anatomical systems; anatomical structures, cell types, biomarker (ASCT+B) tables and two-dimensional (2D) and 3D reference objects), experimental data mapped to the HRA, as well as enriched atlas data in support of different atlas applications. The origin and evolution of HRA and ASCT+B tables are detailed in previous work^3^. The CCF provides quantitative workflows for integrating new experimental data into the growing atlas, such as histology images, vascular pathways and single-cell analyses. The resulting HRA provides data evidence for common states of cells and anatomical structures in the human body at specific 3D locations and this can be used as a canonical reference to describe the changes that occur across biological variables (for example, age, sex, race and body mass) and acute or chronic diseases. It can benefit applications such as drug development by providing a better understanding of perturbations of cell types and states in diseased conditions, which could reveal relevant targets for precision medicine through comparisons of diseased to non-diseased tissue, and a CCF-matched reference.

When HuBMAP was launched, the first unifying concepts that map major organs in the human body across scales were emerging^4,5^. Existing atlases used organ-specific references (for example, Waxholm Space for the brain^6^ or the Helmsley one-dimensional distance reference system for the colon), but most of these references do not map to a shared human body CCF^7^. To advance CCF development, in March 2020, the National Institutes of Health (NIH) and the Human Cell Atlas (HCA)^8^ Consortium organized a joint virtual meeting with a CCF breakout session. This resulted in the formation of the HRA Working Group (WG). Over the last 55 months, WG members jointly developed a definition and key properties for the HRA. These properties are:

1. **The HRA defines a reference 3D multiscale space and shape of anatomical structures and cell types and the biomarkers used to characterize cell types**. Anatomical structures, cell types and biomarkers are validated against, or are added to, existing ontologies (for example, the uber-anatomy ontology (Uberon)^9^, the Foundational Model of Anatomy Ontology (FMA)^10,11^, Cell Ontology (CL)^12^, Provisional Cell Ontology (PCL)^13^ and the Human Gene Ontology Nomenclature Committee (HGNC; https://www.genenames.org)). As more data are collected, the HRA will increasingly be able to show how body shape and size, plus cell type populations differ across individuals and change over a person’s lifespan.
2. **The HRA enables adding new experimental datasets and mapping these to existing data through a variety of mechanisms**. For example, the location of tissue specimens can be specified relative to virtual 3D reference organ models in the HRA; single-cell genomic data can be mapped to the HRA using annotation tools like Azimuth^14^; and, single-cell-resolution spatial proteomics data can be mapped to the HRA using validated organ mapping antibody panels (OMAPs)^15^. With the development of new technologies and computational methods in the future, additional mappings and linkages, such as integration of multi-omics data, will be possible.
3. **The HRA follows best practices and standards for sharing scientific data**. To do this, the HRA needs to be authoritative (it should be supported by peer-reviewed scholarly publications, experimental data evidence or expert consensus); meet the Transparency, Responsibility, User focus, Sustainability and Technology (TRUST) principles for digital repositories^16^; be representative (covering all major human demographics and welcoming everyone to contribute to and use the HRA data); be open and adhere to the Findable, Accessible, Interoperable, Reusable (FAIR) principles^17^ (where anyone can use the HRA data and code and these are provided in community standard formats with linked ontologies; be published as linked open data (LOD) connected to ontologies and other LOD; and application programming interface (API) queries and user interfaces are supported); detailed protocols and standard operating procedures (SOPs) are provided; and be continuously evolving (for example, as new technologies, data and methods become available).

Experts also agreed on key SOPs for atlas construction and usage plus HRA terminology (Box 1), adopted from the HRA SOPs Glossary^18^. Plus, the HRA WG brought together technical leads from the HuBMAP Integration, Visualization & Engagement (HIVE) Collaboration with experimental teams in HuBMAP and experts from the Genotype-Tissue Expression (GTEx)^19^, GUDMAP: GenitoUrinary Developmental Molecular Anatomy Project^20^, Kidney Precision Medicine Project (KPMP)^21,22^, LungMAP^23,24^, BRAIN Initiative Cell Census Network Initiative (BICCN)^25,26^, Cellular Senescence Network (SenNet)^27^ and other NIH-funded consortia and with strong support from the HCA effort^8,28^ to develop the HRA data, code and portal infrastructure together.

An important next step in the HRA effort was collecting user stories in support of atlas construction and usage to encourage dialog, deliberation and iteration by designers and users around three key questions: Who are the users involved in a particular user story? What are the outcomes they hope to achieve? What value do they stand to gain? Agreement on answers to these questions helped prioritize user needs, provided context for proposed user stories and reduced ambiguity.

More than 30 one-on-one interviews were conducted with atlas architects (experts who serve as principal investigators or are otherwise intimately involved in the construction of the latest generation of human atlases, including BICCN, GTEx, GUDMAP, HCA, HuBMAP, Human Tumor Atlas Network (HTAN)^29^, KPMP, LungMAP, (Re)building the Kidney (RBK)^30^ and SenNet). Given the interdisciplinary nature of this effort, the atlas architects who were interviewed comprise a diverse group of physicians, laboratory and computational biologists, engineers and computer and data scientists. In addition, six programmers from different human atlas projects were surveyed.

The interview and survey results helped identify three key objectives and seven concrete user stories (US 1–7) for the construction, usage and sustainability of the HRA (Table 1). The three objectives are:

1. The HRA should facilitate atlas construction by aligning new tissue blocks with existing data. For example, developers of the HRA want to predict cell type populations for new tissue blocks (US 1 in Table 1) and predict the spatial origin of tissue samples with known cell type populations (US 2).
2. The HRA should contain functionality that provides insights into changes (for example, with aging, disease or other perturbations) that occur at all levels in the body. To do this, researchers and clinicians need to be able to search for and explore cell types and biomarker expression values for tissues and functional tissue units (FTUs) (US 3 and US 4) and determine the location and distances between cells (US 5).
3. The HRA should use processes that encourage collaboration and guide future development to ensure long-term sustainability. This includes leveraging architectures with modular, lightweight components that can be easily shared (US 6) with metrics of success provided via an HRA dashboard to researchers, clinicians and funders to gain feedback and support (US 7).

**Table 1:** User stories. Feature summary, target user roles, user activities and added value for seven user stories that drive HRA development

Feature	User role	User activities	Added value
**Facilitate atlas construction by aligning new tissue blocks with existing data**			
US 1. Predict cell-type populations US 2. Predict spatial origin of tissue samples	Programmers that support researchers, clinicians, pathologists Programmers that support Researchers, clinicians	Predict and explore the likely cell type populations for a RUI-registered tissue block. Predict and explore the likely 3D location in the human body for a given tissue block with known cell type population.	Improve cell type annotation through information on what cell type populations exist in what anatomical structures. Compensate for the absence of spatial origin information in many single-cell datasets.
**Use the atlas to gain insights into changes that occur at all levels in the body with aging or disease**			
US 3. Compare reference tissue with aging/diseased tissue US 4. Compare reference FTU with aging/diseased FTUs US 5. Provide cell distance distribution visualizations	Researchers, clinicians Researchers, clinicians Researchers, pathologists	Compare tissue blocks, cell types and biomarker expression levels between healthy reference tissue and aging/diseased tissue. Compare FTUs in terms of cell types and mean biomarker expression levels for healthy reference tissue and aging/diseased tissue. Compute, visualize and explore distance distributions between different cells, cell types and anatomical structures (for example, FTUs) and cell types and morphological features (for example, the edge of an organ).	Understand and communicate changes in tissue structure and function with age or disease. Understand and communicate changes in FTU structure and function with age or disease Add granularity to our understanding of how disease develops (for example, how tumor cells grow or metastasize) in support of targeted therapies.
**Ensure atlas sustainability with processes that encourage collaboration and guide future development**			
US 6. Develop lightweight atlas components US 7. Implement dashboard for HRA	Programmers that support researchers and clinicians Researchers, clinicians, funders	Implement usable and useful HRA components (interfaces and APIs) into other portals in the growing ecosystem of human atlases. Track the evolution and usage of the HRA using data, code and portal usage statistics in aggregate and divided by portal (for example, HuBMAP or SenNet) or user survey results.	Facilitate collaboration and data/code reuse between the HRA and other portals in support of FAIR data principles. Enable evidence-based decision-making by providing insights into the atlas’ construction and usage (for example, gaps in data, application areas, user demographics, equitable access).

**Fig. 1:**
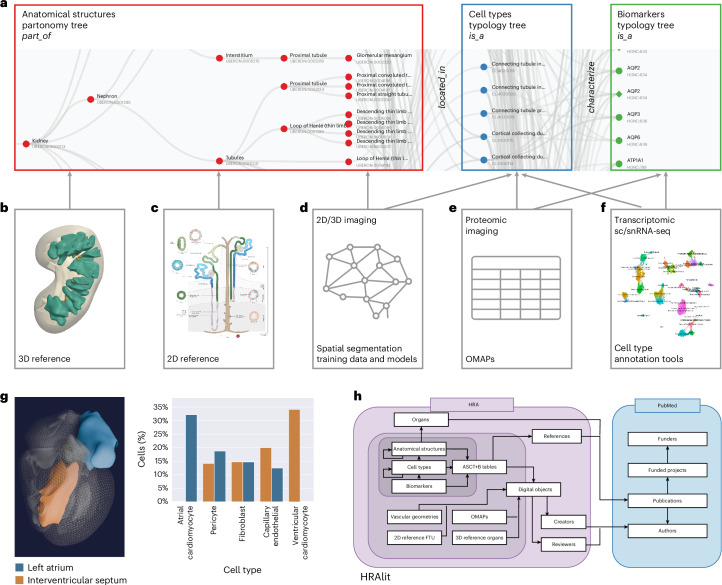
Human Reference Atlas components and linkages. **a**, The ASCT+B tables document the nested ‘part_of’ structure of organs (for example, cells that make up FTUs, successively larger anatomical structures, an entire organ such as the kidney, which is part_of the body). The cells that make up (are ‘located_in’) each of the anatomical structures are organized in a multilevel cell-type typology with ‘cell’ at the root and more and more specialized child nodes and ‘is_a’ relationships between nodes. The biomarkers used to characterize cell types might have one of five types: genes, proteins, metabolites, proteoforms and lipids organized in a biomarker typology. Gray arrows indicate crosswalks that connect other HRA DOs to ASCT+B tables. **b**, HRA 3D reference objects represent the shape, size, location and rotation of 1,192 3D anatomical structures with 516 unique Uberon IDs for 65 organs with crosswalks to ASCT+B tables. Shown are ‘renal papilla’ and ‘renal pyramid’ in the kidney. **c**, 2D reference illustrations document the shape, size and spatial layout of 3,742 2D cells of 116 types for 22 FTUs in ten organs with crosswalks to ASCT+B tables. Shown is the kidney nephron. **d**, Labeled training data exist for FTUs in five organs with crosswalks (gray arrows) to anatomical structures and cell types in the ASCT+B tables. **e**, 13 OMAPs are linked to 197 AVRs and there exist crosswalks to cell types and biomarkers in ASCT+B tables. **f**, Ten Azimuth references for healthy adult organs plus crosswalks to cell types and biomarkers in ASCT+B tables. **g**, HRApop reports cell type populations for anatomical structures compiled from experimental data. Exemplarily shown is the left atrium (blue) and the interventricular septum (orange) of the female heart plus a bar graph with the cell types that have the highest percentage across these two anatomical structures (annotated with Azimuth). Note that some cell types appear only in one anatomical structure. **h**, The HRAlit database links HRA DOs to existing ontologies (for example, Uberon and CL), expert ORCID, publication evidence, funding and experimental data used for HRApop computation.

The three key objectives and associated user stories help focus presentations and discussions in the monthly HRA WG; they are driving HRA development and iterative optimization. Every 6 months, a new HRA release is published. With every release, existing ontologies are expanded plus HRA data structures and algorithms are improved to better serve the needs of the international human atlasing community. Figure 1 details major components of the sixth release of the HRA and their interlinkages.

Box 1 Key HRA terminology

- 3D collision: the intersection of 2D or 3D bounding-box volumes or surface polygon meshes.
- 3D model: DO with a shape and size defined by polygon meshes (vertices and edges) that can be used to represent the real-world form of anatomical structures, cells or proteins in 3D.
- 3D Reference Object: polygon mesh of 3D spatial objects (for example, anatomical structures), their object node hierarchy, materials and surface color and texture. They are created by medical illustrators with the involvement of subject matter experts following standard operating procedures.
- Anatomical structure: a distinct biological entity with a 3D volume and shape, for example, an organ, FTU or cell.
- AVR: document providing details on the characterization of individual antibodies for multiplexed antibody-based imaging assays. These details include target protein information (for example, target name, UniProt accession number) and antibody information (for example, RRID, host organism, vendor, catalog number, lot number). AVRs also provide details on controls used for antibody characterization and validation (positive and negative tissues, cell lines, isotype controls, etc.), exemplar imaging data and information on other antibodies tested.
- ASCT+B tables: these are authored by multiple experts across many consortia. Tables capture the partonomy of anatomical structures, cell types and major biomarkers (for example, gene, protein, lipid or metabolic markers) defining cellular identity supported by scientific evidence and are linked to ontologies.
- Atlas-enriched dataset graph: a graph for highest quality datasets used for HRA construction (it has an extraction site, cell type population and publication or provenance on a major atlas portal), enriched with additional metadata, computed by HRApop.
- Biomarkers (B): HRA biomarkers are used to characterize or identify cell types. They include genes (BG), proteins (BP), metabolites (BM), proteoforms (BF) and lipids (BL).
- Cell suspension: single cells or nuclei isolated from a tissue (for example, using enzymes or mechanical means) for single-cell assays, for example, before sc/snRNA-seq assay is run.
- Cell types: tissue is composed of different (resident and transitory) cell types that are characterized or identified via biomarkers.
- CTann: Azimuth and other cell type annotation tools are used to assign cell types to cells from sc/snRNA-seq studies. Manually compiled crosswalks are used to assign ontology IDs to CTann cell types.
- Cell type population: a listing of unique cell types, the number of cells per cell type and mean biomarker expression values per cell type computed for anatomical structures, extraction sites and datasets.
- CCF: the HRA CCF consists of ontologies and reference object libraries, computer software (for example, user interfaces) and training materials that (1) enable biomedical experts to semantically annotate tissue samples and to precisely describe their locations in the human body (‘registration’); (2) align multimodal tissue data extracted from different individuals to a 3D reference coordinate system (‘mapping’) and; (3) provide tools for searching and browsing HuBMAP data at multiple levels from the whole body down to single cells (‘exploration’). Alternative CCF definitions do exist^82^.
- Cosine similarity: measures the cosine of the angle between two vectors, with 1 indicating identical vector directions and 0 indicating no similarity.
- Crosswalk: an ontological mapping of terms in HRA DOs (for example, 2D/3D reference objects, OMAPs and Azimuth references) to ontology terms in the ASCT+B tables.
- Dataset graph: a JSON-LD file containing a graph of RUI registration, donor, experimental (for example, links to cell by biomarker (C×B) H5AD files or cell type population), literature and provenance data.
- Digital object (DO): unit of information that includes properties (attributes or characteristics of the object) and may also include methods (means of performing operations on the object).
- Digital object identifier (DOI): centrally registered identifier composed of a string of numbers, letters and symbols used to uniquely identify an article or document with a permanent web address or uniform resource locator (URL).
- Extraction site: digital, 3D representation of a tissue block. If the RUI is used to register tissue, then each site has a unique ID; data on size, location and rotation in 3D in relation to a HRA reference organ; a listing of all anatomical structures that the cuboid intersects with (bounding-box collision by default); and metadata on who registered it.
- FAIR principles: acronym for findable, accessible, interoperable and reusable, which is a way of sharing data to maximize its utility^17^.
- FTU: a small tissue organization (that is, set of cells) that performs a unique physiologic function and is replicated multiple times in an organ. Examples are liver lobule, alveolus of lung or pancreatic acinus.
- H&E stain: histology stain that is widely used as a gold standard for pathological evaluation of tissue sections.
- HRA: the HRA is a comprehensive, high-resolution, three-dimensional atlas of major cells in the healthy human body. The HRA provides standard terminologies and data structures for describing specimens, biological structures and spatial positions linked to existing ontologies.
- HRAlit: scholarly publication linked to HRA DOs to provide scholarly evidence.
- HRApop: experimental data linked to HRA DOs to provide data evidence and number of cells per cell type per 3D anatomical structure.
- LOD: a method for sharing data in standard, non-proprietary formats and deeply interlinked with other data resources.
- Millitome: a device used to hold and slice organs into a grid of equally sized tissue blocks plus a process of generating HRA-aligned digital tissue blocks.
- Ontology: a set of subject area concepts (here, anatomical structures, cell types and biomarkers), their properties and the relationships between them. Ontologies used in the HRA include Uberon and Cell Ontology (CL).
- OMAP: a comprehensive panel of curated antibodies that identifies the major anatomical structures and cell types in a specific organ. The selected antibodies are optimized for a tissue preservation method (fixed or frozen) and multiplexed imaging modality (for example, CODEX and Cell DIVE) through published protocols (protocols.io) and AVR.
- Partonomy: a classification hierarchy that represents part-whole relationships.
- Polygon mesh: a collection of vertices, edges and faces defining the shape of a polyhedral object, for example, tissue block cuboids or reference objects denoting anatomical structures.
- Reference objects: a 3D model of anatomical structures created by medical illustrators with the involvement of subject-matter experts following SOPs.
- Registration set (see also dataset graph): grouping of tissue blocks by the study/paper in which they were published. Each set has a human-readable registration set name, a machine-readable internationalized resource identifier and it links to a paper DOI and tissue block ID.
- RUI registered: tissue spatially and semantically registered to the HRA using the RUI.
- Segmentation: image processing that predicts boundaries of objects, for example, anatomical structures such as nuclei, cellular membranes, cells or FTUs in 2D or 3D.
- Single-cell transcriptomic data (sc/snRNA-seq): data from single-cell (sc) or single-nucleus (sn), high-throughput suspension-based studies that measure polyadenylated RNA molecules in an individual cell.
- Single-cell proteomic data: data from single-cell studies using CODEX, Cell DIVE, Ibex, CycIF or other assays that detect proteins in situ in a tissue, consequently enabling protein expression quantification.
- Tissue block: a sample or specimen derived from an organ or tissue including subsections thereof obtained from a donor that has a unique ID and links to donor organ extraction site, processing, preservation and other metadata. The locations of tissue blocks are registered using the RUI.
- Tissue section: a thin (several-μm) section of a tissue block usually obtained using a cryotome or microtome. Tissue sections inherit the location and rotation of their parent tissue block. The thickness and number of tissue sections is captured in an input field inside the RUI.
- Typology: a classification that represents general types, for example, cell types or biomarker types.
- United file: a GLB 3D object that contains all the modeled organs in the HRA.
- VCCF: a proposed approach to use vasculature as a coordinate system to map all the cells in the human body.
- Web Ontology Language (.owl) file format: the World Wide Web Consortium (W3C) Web Ontology Language is a Semantic Web language (documented at https://www.w3.org/OWL).

## Results

The HIVE Infrastructure and Engagement Component (IEC) developed HuBMAP’s flexible hybrid cloud microservices architecture (Supplementary Fig. 1 and Methods) to support data curation, ingestion, integration, access, analysis, exploration and download via the HuBMAP Data Portal (https://portal.hubmapconsortium.org). HIVE Tools Components focused on the HuBMAP Data Portal User Interface, visualization, workflow integration and tool development. HIVE Mapping Components developed Azimuth^14^ references and the HRA Portal (https://humanatlas.io) in close collaboration with external experts.

The HuBMAP Consortium website (Supplementary Fig. 2) provides easy access to HuBMAP resources, publications, news, internship programs, member services, etc. It links to the HuBMAP Data Portal and the HRA Portal. The HuBMAP Data Portal provides access to HuBMAP data, APIs and user interfaces with continuous data and code releases. The HRA Portal serves atlas-level data and code created by 18 atlas projects and new HRA releases are published every 6 months. Both portals use knowledge graphs (KGs) to store data and the HRA KG is regularly ingested into the Unified Biomedical Knowledge Graph (UBKG; https://ubkg.docs.xconsortia.org) to link HuBMAP experimental data to existing ontologies and the HRA. The HRA uses HuBMAP and other experimental data to compute cell type populations for anatomical structures (see HRA cell type populations (HRApop) in Methods and Supplementary Table 1). Several HRA user interfaces (see section User interfaces) are deployed in the HuBMAP Data Portal and other portals to support HRA construction and usage.

Atlas construction is complex and requires community agreement on data formats, APIs and user interfaces. Previews are used to showcase and optimize new functionality before it is integrated into the HuBMAP or HRA Portal. Primary data repositories are listed in Supplementary Table 2 and HRA code repositories in Supplementary Table 3.

### Flexible hybrid cloud infrastructure for HRA and HuBMAP

Systematic integration of more than 50 open-source algorithms developed by more than 30 teams is non-trivial. Agreement on metadata and API calls is required to make the output of one algorithm compatible with the input expected by the next (set of) algorithms. Several algorithms crucial to tissue segmentation and annotation were developed by biologists with deep subject-matter domain expertise but limited knowledge on how to build production pipelines. The HIVE production development team worked closely with the original algorithm authors to package their algorithms in a way so that they can be run reliably at scale in a hybrid cloud infrastructure that is flexible and extendable to meet evolving needs.

Specifically, the HIVE IEC, composed of members from the Pittsburgh Supercomputing Center (PSC), the University of Pittsburgh (Pitt) and Stanford University, implemented a flexible hybrid cloud infrastructure and community engagement platform supporting delivery of HuBMAP’s vision in the following key areas: (1) curation and ingestion: semi-automated data ingestion (https://software.docs.hubmapconsortium.org) currently from HuBMAP data providers and (in the future) from community partners and the general research community, to maximize efficiency and usefulness for building the HRA; (2) integration: automated analysis and annotation of ingested data and alignment of these annotations to the HRA via the UBKG; (3) findability and accessibility: manifestation of backend resources in the modular architecture of APIs and containers, services and documentation (https://software.docs.hubmapconsortium.org) that minimizes user friction in integrated searching, querying, analyzing and viewing of HuBMAP data and in the future of tissue maps at multiple spatial scales and among multiple layers of information; (4) interoperability: use of the HuBMAP deployment of the UBKG with extensions to create the HuBMAP Ontology API (https://smart-api.info/ui/d10ff85265d8b749fbe3ad7b51d0bf0a) to translate HuBMAP data, HRA assets and community data among one another via ontologies; the HuBMAP Ontology API contains end points for querying a UBKG instance with content from the HuBMAP context (https://ubkg.docs.xconsortia.org/contexts/#hubmapsennet-context); (5) analysis: infrastructure support to currently enable users with interactive analyses of HuBMAP data via Jupyter notebooks and in the future, batch workflows among both HuBMAP and user-contributed data and tools, including integration and mapping against the HRA; and (6) sustainability: HuBMAP’s flexible hybrid cloud infrastructure (efficiently leveraging on-premises resources at PSC for services that would incur much higher public cloud charges compared to on-premises, such as data storage, processing, analysis and download (Supplementary Fig. 1 and Methods)) will facilitate sustainability of open tools, data and infrastructure beyond the end of the HuBMAP program.

### Atlas construction and publication

HRA data comprises human-expert-generated data (for example, ASCT+B tables, OMAPs, antibody validation reports (AVRs) and 2D/3D reference objects), experimental data mapped to the HRA (via registration user interface (RUI) location, HRA-aligned cell type annotation (CTann) or OMAP/AVR) and enriched atlas data (for example, HRApop and HRA literature (HRAlit)); see Fig. 1 for an overview of HRA digital object (DO) types and their crosswalks (see Box 1 for terminology and Methods for details). HRA data, usage and extension of ontologies, unified data processing workflows, user interfaces, documentation and instructional materials are detailed here.

#### Data types and status

The sixth release of the HRA v.2.0 (December 2023) includes an anatomical structure systems graph which groups major organs into organ systems (for example, digestive system and reproductive system); three ASCT+B tables that represent the branching structures for the blood and lymph and the peripheral nervous system; and 29 ASCT+B tables that document the nested ‘part of’ structure of other organs (for example, kidney with cells that compose smaller and the subsequently large FTUs and organ parts) for a total of 33 ASCT+B tables. The cells that make up each of the anatomical structures are organized in a multilevel cell type typology, with ‘cell’ at the root and successively more specialized child nodes. Cells are mapped to five biomarker types: genes, proteins, metabolites, proteoforms and lipids organized in a biomarker typology.

Anatomically based 3D reference objects (Fig. 1b) in the HRA include the shape, size, location and rotation of 1,192 3D anatomical structures with 516 unique ontology IDs in 65 organs. A SPARQL query (https://apps.humanatlas.io/api/grlc/ccf.html#get-/as-3d-counts) returns all anatomical structures with an Uberon ID (it retrieves the 1,192 anatomical structures plus the 65 organs for a total of 1,257 items). 2D references (Fig. 1c) describe the spatial layout of 3,742 rendered 2D cells of 116 unique cell types for 22 FTUs in 10 organs. Labeled training data for spatial segmentation and machine-learning models (Fig. 1d) exist for five FTUs in five organs. A total of 13 OMAPs (Fig. 1e) are linked to 197 AVRs and aligned with ASCT+B tables. Cell-type annotation tools (Fig. 1f) include Azimuth and other references for healthy adult organs with crosswalks to cell types and biomarkers in the ASCT+B tables.

An important part of HRA processing is data enrichment. One example is HRApop (Fig. 1g), which covers 553 tissue datasets that are used to compute cell type populations for 40 anatomical structures for which 3D reference objects exist, across 23 organs with 13 unique Uberon IDs. The code to reproduce the bar graph with HRApop data (seven datasets) is available^31^. HRAlit^32^ (Fig. 1h) links HRA DOs to 7,103,180 publications, 583,117 authors, 896,680 funded projects and 1,816 experimental datasets.

#### Data enrichment

This HRA processing step ensures that HRA DOs are high quality, usable and useful for the user stories listed in Table 1 and other applications. Normalization ensures that the raw data are well structured and presented in a format that can be readily translated into a knowledge graph via LinkML (https://linkml.io). During enrichment, certain implicit relationships are made explicit using OWL reasoning (for example, transitive relationships like subclass and ‘part of’ are made explicit); external metadata are added from ontologies via APIs to enhance the graph’s usefulness (for example, via queries to the scicrunch API to look up antibody information for OMAPs); queries are used to add data from related graphs (for example, extracting additional metadata and hierarchies related to anatomical structures, cell types and biomarkers from popular biomedical ontologies like Uberon and Cell Ontology); and finalizes conversion from LinkML to knowledge graph (for example, converting and combining all into an RDF-formatted graph in Turtle format).

#### Data publication

A new revised and extended version of the HRA DOs together with updated user interfaces and APIs are published every 6 months via the HRA Portal (https://humanatlas.io). The three HRA core ontologies (specimen, biological structure and spatial ontologies)^7^ are shared as FAIR, versioned LOD at https://lod.humanatlas.io. Select data are also provided in a relational database and as comma-separated value (CSV) files. RUI data are published via the HuBMAP, SenNet, GUDMAP, GTEx and other portals. For instance, the HuBMAP Search API is queried by the HRA API to generate dataset graphs from HuBMAP data. The public graph with all donor, tissue block, tissue section, RUI data and experimental dataset information can be accessed via the HRA dataset graph at https://lod.humanatlas.io/ds-graph.

The HRA DO processor (https://github.com/hubmapconsortium/hra-do-processor) supports automated processing of HRA data, including data normalization, validation, graph transformation, enrichment and publishing. The end product is the HRA KG (https://github.com/hubmapconsortium/hra-kg) and a set of flat files suitable for hosting all data as LOD. HRA infrastructure is optimized for deployment to Amazon S3, Amazon Web Services (AWS) AppRunner and AWS CloudFront, but could be adapted to other file hosting platforms.

The HRA provenance graph keeps track of all HRA DOs (described using standard terminology from DCAT (https://www.w3.org/TR/vocab-dcat) for organizing catalogs of data and W3C-Prov (https://www.w3.org/TR/prov-overview) for describing the provenance of any particular piece of data) and code versions (via GitHub) so HRA KG provenance can be accessed and the HRA KG can be recomputed every 6 months.

Supplementary Table 2 lists all data used in HuBMAP Data Portal (H), HRA Portal (A) and demonstration Previews (P). Note that HRA data are mirrored by the European Bioinformatics Institute’s (EBI’s) Ontology Lookup Service (OLS), Stanford University’s NCBO BioPortal and University of Michigan Medical School Ontobee. Publishing the HRA via widely used repositories for biomedical ontologies makes the HRA FAIR; users can browse the HRA data online or access it programmatically via APIs.

#### Usage and extension of ontologies

Data and workflows are linked to existing ontologies whenever possible (Table 2). The sixth release of the HRA v.2.0 uses biological structure ontologies Uberon 2023-10-27 (ref. ^9^) and FMA v.5.0.0 (refs. ^10,11^) for anatomical structures; Cell Ontology (CL) v.2023-10-19 (ref. ^12^) and PCL 2023-02-27 (ref. ^13^) (https://www.ebi.ac.uk/ols4/ontologies/pcl) for cell types; HGNC v.2023-09-18 (ref. ^33^), Ensembl Release 111 (ref. ^34^), GeneCards v.5.19: 15 January 2024 (refs. ^35,36^) and UniProt Release 2024_1 (ref. ^37^) for biomarkers. The Human Genome HGNC v.2023-09-18 is used for the FTU Explorer. Spatial data are annotated using Dublin core terms (DCTERMS) v.2020-01-20 (https://www.dublincore.org). Specimen data use LOINC v.2022-07-11 (v.2022AB)^38^ for standardized representation of sex, race and ethnicity data. Meta-ontologies such as DCTERMS and Relation Ontology^39^ (RO) are used to capture relationships among concepts within the HRA data. Assay type names come from BioAssay Ontology (BAO) v.2023-01-31 (ref. ^40^) and Experimental Factor Ontology (EFO) v.2023-02-15 (ref. ^41^). The use of these ontologies is strongly encouraged to maintain consistency among ASCT+B tables, Azimuth and other CTann tools and OMAP data in support of atlas construction and usage.

**Table 2:** Ontologies used and extended

DO type	Ontology name and version	No. terms added/updated	No. links added/updated
**Specimen**			
	LOINC v.2022-07-11 (v2022AB)		
**Biological structure**			
Anatomical structures Cell types Biomarkers Human genome	Uberon v.2024-09-01 CL v2024-09-26 PCL 2024-07-12 HGNC v2023-09-18 Ensembl Release 111 UniProt Release 2024_1 GeneCards v.5.19: 15 January 2024 HGNC v.2023-09-18	125 new terms added to Uberon through Oct 2024 141^b^ new terms added to CL through Oct 2024 468 new terms added to PCL through Oct 2024 None None	609 relationships added to Uberon through Oct 2024 294 Uberon terms assigned 3D reference objects^a^ 302 relationships added to CL through Oct 2024 468 relationships added to PCL through Oct 2024 None None
**Spatial**			
Terms Relations	DCTERMS v.2020-01-20 Relation Ontology used by Open Biological and Biomedical Ontologies	None None	None None
**BioAssay and Experimental Factor types**			
BioAssay Experimental Factor	BAO v.2023-01-31 EFO v.2023-02-15	None	None

^a^The foaf:depiction annotation is used to link 294 unique Uberon terms to 586 GL transmission binary (GLB) files.

^b^25 out of 129 terms and 37 out of 249 links were added to CL supported by projects other than HuBMAP.

A major contribution of the cross-consortium HRA effort is the extension of cross-species ontologies such as Uberon and CL to cover healthy human terms. Between 2021 and October 2024, 125 anatomical structure terms have been added to Uberon, 141 cell types were added to Cell Ontology. By October 2024, 468 cell types were added to the PCL, 461 for the human brain^42^ (in support of HRA construction and usage). PCL uses computationally derived marker genes from NS-Forest^43^ to define sc/snRNA-seq-derived cell types in the brain. The 461 human brain cell types were added to the ASCT+B tables. All PCL cell type terms are associated with biomarker genes using a has_characterizing_markerset relation in the ontology. In the sixth release of the HRA, there are 962 anatomical structure terms that are either missing from Uberon or not yet crosswalked to Uberon terms in the ASCT+B table. The majority of missing terms are for blood and lymph vasculature, skeleton or skeletal muscle systems and are typically more specific than currently represented in Uberon (for example, ‘dorsal branch of lateral proper palmar digital artery of fifth digit of hand’). Work is ongoing to improve mappings (~100 mappings were recently added and will be published in seventh HRA release). A total of 119 cell types are either unmapped or not yet in CL or PCL (an initial assessment suggests 60) and 70% of these are genuinely new terms for CL. These 387 biomarkers have Ensembl IDs or GeneCards IDs or have not been mapped rather than HGNC IDs (all of these terms have ASCTB-TEMP IDs). There are GitHub issues to add new terms to existing ontologies to properly represent data in the ASCT+B tables, including requests for 128 anatomical structures in Uberon. There now exists a formal operating procedure to include new cell types into CL via Minimal Information Reporting About a CelL (MIRACL) sheets^44^. The number of ontology relationships added to Uberon, CL and PCL is listed in the last column in Table 2.

#### Unified processing workflows

**Fig. 2:**
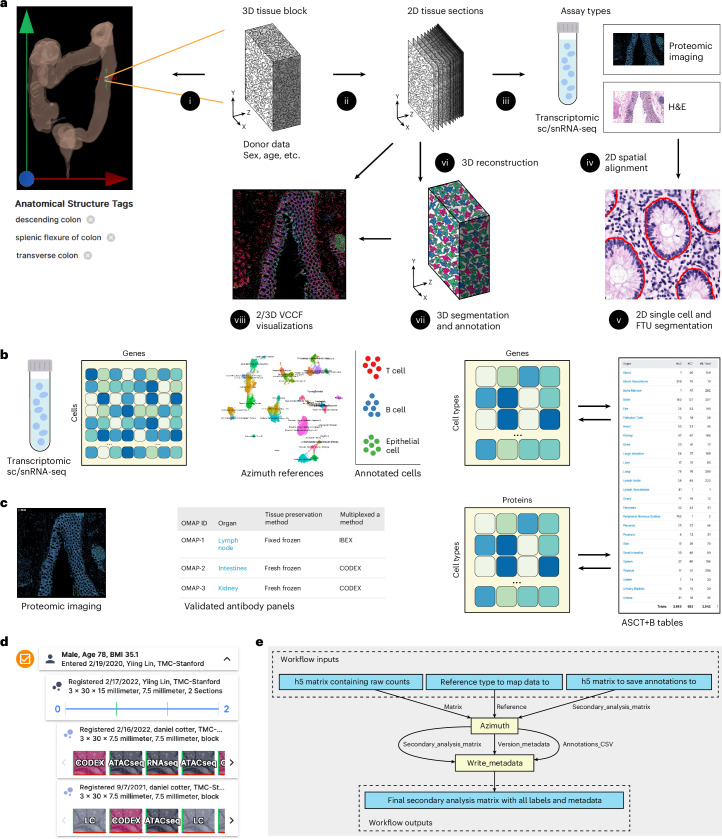
Mapping experimental data to the HRA. **a**, A tissue block is 3D spatially registered and semantically annotated using the RUI or millitome (i). A smaller part of the tissue block might be used for sc/snRNA-seq analysis (not shown) or cut into tissue sections (ii). Tissue sections are analyzed using one or more assay types (iii). Shown are single-cell transcriptomics (for example, sc/snRNA-seq), OMAP-aligned spatial proteomics (for example, CODEX and Cell DIVE) and high-resolution hematoxylin and eosin (H&E)-stained histology images. Spatial alignment of different assay types for the very same or different tissue sections is non-trivial (iv). H&E data are used to segment FTUs using trained machine-learning models (v). A 3D reconstruction of tissue volumes is accomplished by aligning data from multiple serial tissue sections computationally (vi) followed by 3D segmentation and annotation (vii). The 2D or 3D data are analyzed to identify the distance of different cell types to the vasculature (VCCF visualizations) as a multiscale CCF from which no other cell is very distant (viii). **b**, Single-cell/nucleus data (sc/snRNA-seq) is stored as a cell-by-gene matrix; cell types are annotated using Azimuth or other cell type annotation tools; results are aggregated to cell-type-by-gene biomarker expression value matrices that are aligned with the ASCT+B tables; and are used in diverse HRA user interfaces (for example, EUI and FTU Explorer). **c**, OMAP-aligned spatial data generated using validated antibody panels linked to AVRs are analyzed to compute cell type by protein biomarker expression value matrices that are aligned with the ASCT+B tables using semi-automated workflows. **d**, The EUI provides full provenance for donors (sex, age and body mass index), data providers (upload date, contact name and affiliation), tissue blocks and sections (size, number, date and contact info for RUI registration) and datasets (assay type) with links to raw data in the HuBMAP Data Portal, other data portals or publications. **e**, CWL workflows detail which tools (yellow) are run on which input/output data (blue). Shown is the Azimuth cell type annotation workflow.

The HRA SOPs^45^ detail the human expert and algorithmic steps needed to construct the HRA and to use it properly. Protocols published on protocols.io and other places are used to compile experimental data in a reproducible manner. In January 2024, there existed 235 HuBMAP protocols^46^ (many of these document the reproducible workflows required to generate data used in HRA construction). Figures 1 and 2 provide an overview of the numerous steps required to construct the HRA and to map new experimental data to it.

The HuBMAP Consortium has developed uniform computational processing pipelines for multiple data types: single-cell (sc)/single-nucleus (sn) RNA-seq, sc/snATAC-seq, multiplexed antibody-based spatial proteomics (CODEX (recently renamed to PhenoCycler) and Cell DIVE), multiplexed ion beam imaging (MIBI), Slide-seq and Visium sequencing spatial transcriptomics and fluorescence in situ hybridization spatial transcriptomics, among others. HuBMAP computational pipelines are all open source, published on GitHub as CWL workflows wrapping tools in Docker images (also executable via Singularity), with supplementary data (genome indexes/annotations and deep-learning models) built into the published Docker images for portability and reproducibility.

The HuBMAP sc/snRNA-seq pipeline (https://github.com/hubmapconsortium/salmon-rnaseq, also used for sequencing spatial transcriptomics such as Slide-seq and Visium), is built on the Salmon quasi-mapping method^47^ and performs gene expression quantification for intronic and exonic sequences, with downstream analysis using Scanpy^48^ and RNA velocity computation via scVelo^49^. Outputs of the sc/snRNA-seq pipeline are annotated with an automated version of the Azimuth cell type annotation tool for supported tissues; these currently include heart, lung and kidney, with additional annotations computed as new Azimuth references are integrated into HuBMAP processing infrastructure.

HuBMAP imaging pipelines (Methods) are end-to-end analysis methods that accept raw images, perform illumination correction, background subtraction and tile stitching if necessary, then perform cell and nucleus segmentation, writing expression and segmentation mask images as multichannel OME-TIFF files. The expression and mask images are further processed via spatial process and relationship modeling (SPRM)^50^, which computes image and segmentation quality metrics using the CellSegmentationEvaluator tool^51,52^, creates cell adjacency maps, computes features for each cell and nucleus, performs unsupervised clustering of cells, nuclei and image pixels, computes biomarkers differentiating one cluster versus the rest for each clustering type and writes results to CSV and HDF5 format for use by end users and in the HuBMAP Data Portal.

For HRApop (Fig. 1g), 445 public datasets from HuBMAP^2,53^, two datasets from SenNet^54^, 91 healthy datasets from two collections from CZ CELLxGENE^55,56^ (‘Cells of the adult human heart’ and ‘LungMAP — human data from a broad age healthy donor group’) and 15 single-cell datasets from GTEx^57,58^ were mapped to the HRA (Methods). As a result, cell-type population data exist for 40 anatomical structures in 23 organs with 13 unique Uberon IDs, separated by single-cell transcriptomics (for example, sc/snRNA-seq) and OMAP-aligned spatial proteomics (for example, CODEX and Cell DIVE). Three organs (large intestine, small intestine and skin) have cell type populations computed from transcriptomics and proteomics data.

For HRAlit^32^ (Fig. 1h), 583,117 experts, 7,103,180 publications, 896,680 funded projects and 1,816 experimental datasets were mapped to the DOs in the HRA (Methods).

#### User interfaces

The HuBMAP Portal (https://hubmapconsortium.org; Supplementary Fig. 2) introduces HuBMAP goals and links to experimental and atlas data, tools and training materials. The HuBMAP Data Portal (https://portal.hubmapconsortium.org) supports ingest, search, exploration and download of experimental data. The HRA Portal (https://humanatlas.io; Supplementary Fig. 3) supports the construction, access, exploration, usage and download of HRA data.

The ASCT+B Reporter^3^ (https://humanatlas.io/asctb-reporter; Supplementary Fig. 4) supports the authoring and review of ASCT+B Tables and OMAPs by human organ experts. Detailed SOPs^45^ and video tutorials^59,60^ exist and more than 170 unique experts have contributed to the HRA as authors and/or reviewers using this tool as measured by the number of unique ORCID IDs listed in relevant DOs of the sixth release HRA.

Azimuth^14^ (https://azimuth.hubmapconsortium.org; Supplementary Fig. 5) was developed by HuBMAP to automate the processing, analysis and interpretation of sc/snRNA-seq and ATAC-seq data. Its reference-based mapping pipeline reads a cell-by-gene matrix and performs normalization, visualization, cell annotation and differential expression (biomarker discovery) analyses (Figs. 1f and 2b). Results can be explored within the app or downloaded for additional analysis. In HuBMAP, Azimuth is used in production mode to automatically annotate sc/snRNA-seq datasets. Crosswalks exist to associate Azimuth cell types to ASCT+B table terms and ontology IDs.

The RUI^60^ (https://apps.humanatlas.io/rui; Supplementary Fig. 6 and SOP^61^) supports the registration of human tissue blocks into the 3D CCF with automatic assignment of anatomical structure annotations that are linked to the Uberon and FMA ontologies based on surface mesh-level collision events. The anatomical structure annotations in combination with ASCT+B table and experimental data make it possible to predict cell types that are commonly found in anatomical structures and colliding tissue blocks. RUI output in JSON format records registration data (for example, tissue block universal unique identifier (UUID) and 3D size, location and rotation plus anatomical structure annotation based on bounding box) together with provenance data (for example, operator name and date). The RUI is available as a stand-alone tool for anyone to use to contribute HRA-aligned spatial data. It is fully integrated in the HuBMAP, SenNet and GUDMAP data ingest portals but requires authentication.

The Exploration User Interface (EUI) (https://apps.humanatlas.io/eui; Supplementary Fig. 7) supports visual browsing of tissue samples and metadata at the whole-body organ, tissue and cell levels (Table 1, US 3). In January 2024, 901 human tissue blocks with 4,221 datasets from 351 donors and 19 consortia/studies were RUI-registered into the HRA 3D CCF. Users can filter by donor demographics (for example, sex and age) or data source (for example, consortium/study and technology). They can search for specific anatomical structures, cell types or biomarkers to explore the number of tissue blocks that collide with an anatomical structure but also the cell types located in these anatomical structures or their characterizing biomarkers (according to the ASCT+B tables). Users can also run a 3D spatial search using an adjustable probing sphere, explore details on demand on the right with links to Vitessce^62,63^ visualizations in the HuBMAP Data Portal and links to data and tools in other data portals. The EUI with all HRA data is available as a stand-alone tool that supports exploration of all experimental data that has been mapped to the HRA. The EUI was customized, branded and fully integrated in the HuBMAP, SenNet and GTEx data portals to support exploration of consortia specific data (Supplementary Fig. 8).

Vitessce^62,63^ (http://vitessce.io) is a tool used to visually explore experimental data, Azimuth references (Supplementary Fig. 5), HRA segmentations and annotations or cell–cell distance distribution visualizations (Supplementary Fig. 9), see previews in the Atlas usage section.

The Interactive FTU Explorer^64^ (https://apps.humanatlas.io/ftu-explorer; Supplementary Fig. 10) supports the exploration of cell types in their 2D spatial context together with mean biomarker expression matrices (Table 1, US 4). For example, tissue data (cell type populations with gene or protein expression levels, as available) can be compared against healthy HRA reference data to determine differences in the number of cells, cell types or mean biomarker expression values to inform clinical decision-making.

The HRA Organ Gallery^65,66^ (https://github.com/cns-iu/hra-organ-gallery-in-vr; Supplementary Fig. 11) supports the multiscale exploration of 1,192 anatomical structures in the 65 3D Reference Objects of the HRA 2.0. Using a Meta Quest VR device, users select the male or female reference body; they can then select a specific organ and explore it with both hands. To achieve view update rates of 60 frames per second, lower level-of-detail models are used that were derived from the original HRA 3D Reference Objects.

The HRA API (https://humanatlas.io/api/; Supplementary Figs. 12–14) supports programmatic access to all HRA DOs and the experimental HRApop data mapped into it. Users first select an API server and route, input query parameters, then view the query response, see Methods for details.

The HRA dashboard (https://apps.humanatlas.io/dashboard) compares HRA, publication and experimental data to world population data. Supplementary Fig. 15a shows population pyramids by age group of HRA survey respondents and tissue data donors in comparison to world population plus population pyramids by career age for HRA experts and publication authors. Supplementary Fig. 15b features the ethnic composition of survey respondents, HRA tissue donors, HRA experts, paper authors and world population in percentages. The choropleth map in Supplementary Fig. 15c shows the number of paper authors overlaid on a world map. CCF–HRA data dashboards help understand what HuBMAP data have been RUI registered (https://hubmapconsortium.github.io/hra-data-dashboard).

#### Documentation and instructional material

In January 2024, the HuBMAP Data Portal provides access to 8 publications and associated datasets, 50+ technical documents (https://software.docs.hubmapconsortium.org/technical) and links to 235 experimental protocols on protocols.io; the HRA Portal links to 20 SOPs (https://zenodo.org/communities/hra) and to the Visible Human Massive Open Online Course (VHMOOC; https://expand.iu.edu/browse/sice/cns/courses/hubmap-visible-human-mooc) with 39 videos, 4 self-tests and 3 quizzes, 2 hands-on tutorials, plus entrance and exit surveys (Supplementary Fig. 16).

### Previews of Atlas usage

Two exemplary previews demonstrate the usage of atlas data and code developed in HuBMAP for gaining insights into pathology, see user stories that drive HRA construction and usage (Table 1). All data and code are publicly available on GitHub^67,68^ and Dryad^69^. The cell distance distribution code is available via the HRA Portal^70^ in support of Table 1, US 5. Cell type annotations for the CODEX multiplexed imaging dataset of the human intestine are published via Dryad. Full data and code integration into the HuBMAP Data Portal workflows are planned for future releases.

#### Perivascular immune cells in lung

**Fig. 3:**
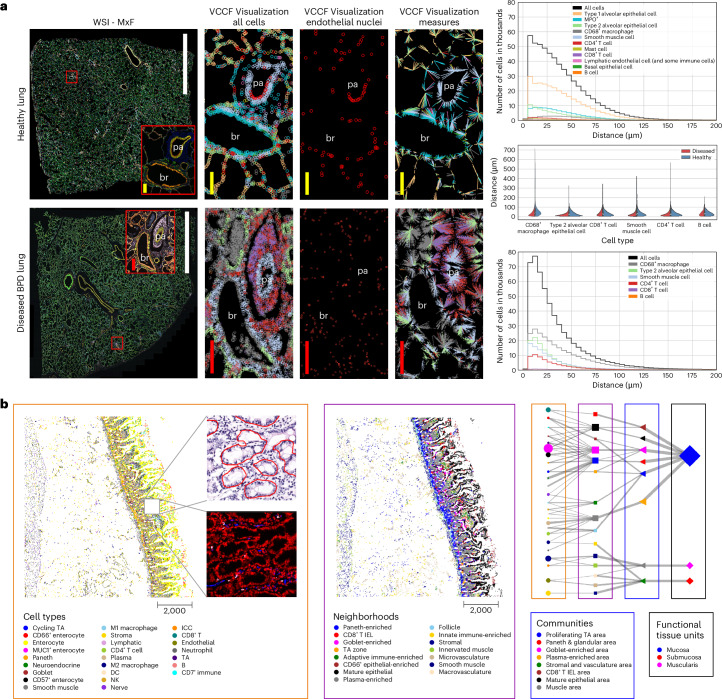
Human Reference Atlas usage. **a**, HRA can be used to compare the distribution of parenchymal cells including endothelial, epithelial and muscle that compose the blood vessels, airways and gas exchanging functional lung structures and resident immune cells including macrophages, to local vasculature (VCCF visualization) in healthy (top) and diseased (bottom) lung using multiplexed immunofluorescence microscopy images with bronchiole (br) and an accompanying small pulmonary artery (pa). Scale bars, white 5 mm; red 200 µm; and yellow 100 µm. The graphs on the right show distance distributions for cell types present in the healthy lung (top) and diseased BPD lung (bottom); the violin plot (middle) shows a comparison between distance distributions for cell types common in both datasets. Datasets are on GitHub^81^. **b**, Multilevel cell neighborhoods can be computed to analyze and communicate the structure and function of FTUs; tissue image with cell type annotations and zoom into H&E with FTU segmentations (red outlines) and zoom into the multiplexed image (CODEX) is shown in left, neighborhoods are given in the middle; hierarchy of FTUs, neighborhoods, communities and cell types are shown on the right. Datasets are on GitHub^81^. ICC, Interstitial cells of Cajal; TA, transit-amplifying; NK, natural killer; DC, dendritic cell; IEL, intraepithelial lymphocytes.

Normal lung function depends on careful matching of airflow to blood flow to achieve normal gas exchange. The abnormal presence and activity of immune cells results in leaky vascular membranes and edema that thickens the gas exchanging membrane and accumulation of mucus and cellular debris in the airspace can cause a mismatch between flow of air and blood. Persistent inflammation results ultimately in fibrosis. Previous work using single-cell RNA sequencing data and the CellTypist common reference dataset discovered previously under-appreciated organ-specific features and aggregates of T cells and B cells^71^. Recent publications in the field of mucosal immunology illustrate the segregation of immune cells within aggregates in human lung tissue and their role in abnormal regulation of vascular function^72–75^. Molecular and cellular changes, including fibrotic and immune-cell-rich regions were recently imaged in the lungs of children with bronchopulmonary dysplasia (BPD), a chronic lung disease following premature birth^76^. The Vitessce tool is used in Fig. 3a (left) to visualize tissue data. Cell distance analyses and visualizations are used to comparatively visualize and quantify cellularity of specific regions of healthy adult and BPD lung to demonstrate an assessment of multiple cell types relative to nearest vascular endothelial cell nuclei using single-cell spatial protein biomarkers.

Demonstrated are whole-slide photomicroscopy images of PhenoCycler^R^ multiplexed immunofluorescence assays (WSI-MxF) applied to examples of healthy lung tissue (top row, 28 antibody panel) and BPD tissue (bottom row, 25 antibody panel). Digital zoom is used to highlight similar regions of interest (red box, MxF-ROI) focused on a bronchiole (br) and an accompanying small pulmonary artery (pa). Immune-cell aggregates, primarily CD3^+^ lymphocytes, are noted around both structures in the BPD lung. To assess a vascular CCF (VCCF) for the localization of the immune and other lung cells, the cell types are masked in cell specific colors (see key) and distances are measured to nearest endothelial cell nucleus (red circles). The cell to nearest endothelial cell measurements are seen as spokes colored by the cell type. In brief, the graphical representations quantitatively demonstrate increased cellularity with a predominance of CD4^+^, but not CD8^+^, lymphocytes, as well as myeloid immune cells, positioned in close proximity to the lung vasculature in the diseased lung. The VCCF visualizations suggest endothelial cells embedded within the lymphocytic aggregates that congregate around the pulmonary artery (pa) in the diseased as compared to the healthy lung. Analyses of cell populations (number of cells per cell type and their mean biomarker expression values), as well as cell to cell and cell to FTU spatial distribution patterns are valuable to understand tissue and cellular disruptions that account for organ failure in lung disease. In this example, the diseased tissue has gas exchange membranes thickened in part by extravascular immune cell aggregates such that distribution of cell distance to nearest endothelial cells is compressed and exaggerated (see graphs in right of Fig. 3a). Measurements on 2D images, as demonstrated, provide novel insights; however, cell segmentation and determination of relative locations in the lung is particularly challenging given the complex airway and vascular branching system and the very thin, highly multicellular gas exchange membranes of the alveoli. It is anticipated that application of similar segmentation, cell to cell and cell to FTU measures to 3D lung tissue volumes will identify currently under-appreciated relationships in health and disease (Table 1, US 5). HuBMAP code can be used on human lung tissue to understand how the spatial organization of specific immune cell infiltrates relate to disease pathophysiology revealing potentials for targeted therapy to ameliorate human disease. Code and data are available at ref. ^68^.

#### Hierarchical cell type populations within FTUs

FTU segmentation algorithms for histology data^77,78^ and hierarchical cell community analysis^69^ for paired spatial data (see Methods for both) can be combined to analyze and communicate the structure and function of FTUs across scales. FTU segmentation algorithms are run as part of the standard HuBMAP workflows (currently limited to glomeruli in kidneys, soon to be expanded to crypts in the large intestine and white pulp in spleen).

Exemplarily, we feature an example hierarchical cell neighborhood analysis previously developed for analyzing cell type neighborhoods across scales and applied within the healthy human intestine^69^ (see Jupyter Notebook on Github^67^). We have named some of these scales: cellular ‘neighborhoods’, ‘communities’ and ‘functional tissue units.’ The calculation of similar cellular neighborhoods, communities and tissue units across different scales is analogous to how we might think that people form neighborhoods, cities and states.

Currently, the HuBMAP Data Portal supports cell segmentation of antibody-based multiplexed imaging data but lacks the ability to annotate the cell types for such datasets. This functionality is under active development (Methods). Consequently, to demonstrate this user story, a separately processed version of the intestine data^79^ (containing cell type annotations) was used. The cell type predictions for the same dataset, using the current development version of the cell type model by the Van Valen laboratory (Methods) are also made available via https://cns-iu.github.io/hra-construction-usage-supporting-information. This version of the model was trained on several multiplexed datasets spanning tissue types and multiplexed imaging modalities. Cell segmentation masks, generated using Mesmer, are also included with the prediction data.

The Jupyter Notebook on GitHub^67^ demonstrates how to read a previously published CODEX multiplexed imaging dataset of the human intestine^69^, identify how cell types correspond to larger multicellular structures and support exploration of the relationships between these higher order cellular neighborhoods. By visualizing the data in tissue coordinates, we can observe potential layering or consistent FTU structures, such as the repeat structure of the intestinal crypt in the proximal jejunum in the small intestine (see Fig. 3b, left). Furthermore, we can quantify these relationships across different scales of cellular neighborhoods and represent them as a network graph (Fig. 3b, right) in which line thickness indicates the percentage of cells in the next level. Note that the tissue samples and the graph use the very same node color coding and naming.

### Usage statistics

Between July 2023 and October 2024, over 33,500 unique users visited the HuBMAP Consortium website (https://hubmapconsortium.org). These users visited 480 distinct pages; the top six most-frequent referrers were Google, pathwaystoscience.org, nature.com, Bing, X/Twitter and psc.edu. Between January 2021 and December 2023, 87,310 unique users visited 382,384 pages in the HuBMAP Data Portal (https://portal.hubmapconsortium.org); the top five most-frequent referrers were nature.com, hubmapconsortium.org, humancellatlas.org, azimuth.hubmapconsortium.org and humanatlas.io. Azimuth supported the upload and cell type annotation of 27,000 datasets with more than 366,000,000 cells. Between June 2023 and October 2024, 1,194,130 HRA Portal requests and 524,358 HRA API requests were fulfilled; the top five referrers were the HuBMAP Entity API, the GTEx Portal (https://gtexportal.org), the HuBMAP Data Portal, the SenNet Data Portal (https://data.sennetconsortium.org/search) and EMBL-EBI (https://www.ebi.ac.uk). The 3D reference objects were accessed 3,065 times via the NIH3D website. The HRA OWL file was accessed 1,325 times via the NCBI BioPortal Ontology Browser (https://bioportal.bioontology.org/ontologies/CCF) and 11,531 times via the EBI OLS Ontology Browser (https://www.ebi.ac.uk/ols/ontologies/ccf). A total of 310 students registered for the VHMOOC and spent 5,652 h reviewing materials, taking self-tests and engaging in a community of practice.

## Discussion

This Resource paper describes data, code and tools that are of broad utility, interest and significance to the construction and usage of a multiscale HRA. The HRA effort and evolving data and code infrastructure is novel and unique in several ways: (1) The HRA integrates many assay types across scales, from whole human body to single-cell level. (2) It provides SOPs and tools to spatially and semantically register human tissue from 65 organs into one CCF. (3) It links anatomical structures, cell types and biomarkers to ontologies and extends existing ontologies when needed. (4) The HRA comes with diverse interfaces that allow users to explore and inspect diverse HRA DOs (3D reference objects, ASCT+B tables, OMAPs, etc.), experimental data and documentation from the participating consortia, as well as HuBMAP data in particular (HRA Portal, ASCT+B reporter, RUI, EUI, Cell–Cell Distance Distribution Visualizations, Interactive FTU Explorer and HRA Organ Gallery in VR). For each user interface, we provide Supplementary Figs. 3, 4, 6, 7 and 9–11) with high-resolution screenshots and detailed annotations. (5) HRA development is community driven and collaborative; monthly WG meetings inform strategic decision-making; 50+ open-source algorithms developed by 30+ teams have been systematically integrated into a flexible and adaptive system architecture that adds value to many atlasing projects; new HRA data and code are publicly released every six months via the HRA Portal and ontology services. The resulting HRA is a multiscale, multimodal, 3D digital product that unifies biomedical knowledge across organs, anatomical structures scales, demographic markers, assay types and links them to ontologies and makes human reference data computable.

The sixth release HRA has several known limitations that will be addressed in future iterations. Starting with the eighth release (published in mid-December 2024), all HRA DOs and their complete provenance are covered in the HRA KG. Cell states are not currently captured in CL nor are ever specific cell types emerging from single-cell technologies; however, the HRA started to use cell type annotation that have the format ‘CL ontology term:cell state or specificity terminology’ (for example, ‘pancreatic stellate cell:quiescent’ mapped to ‘pancreatic stellate cell CL:0002410’, which is in CL or ‘enterocyte:MUC1 positive’ mapped to ‘enterocyte CL:0000584’) along with a confidence term to the CL match (for example, skos:narrowMatch (for cell states or new cell types) or skos:exactMatch (those that exactly match a cell type in CL)) to allow it to be represented in the HRA KG and UBKG and updated at a later date when the computational community has settled on a method to ontologically represent such cells. When there is no exact match, terms in the HRA KG will be given an ASCTB-TEMP ID and individual cells annotated by a cell type annotation model will be given a cell type ID to facilitate future updates when they become available.

In addition, existing workflows for mapping new experimental data to the HRA will be expanded in three main ways: (1) HuBMAP plans to add several new Azimuth references (for example, for large and small intestine) and update existing references (for example, for kidney and lung) to capture new data with additional/revised cell type annotations and CL terms plus CL IDs via crosswalks; (2) eight new OMAPs were published in the seventh HRA release and several more are in progress for the eighth HRA release, substantially increasing the number of spatial datasets that can be mapped to the HRA; and (3) starting with the eighth HRA release, new 3D organs will be added to the RUI: quadriceps femoris and triceps surae skeletal muscles, esophagus and lymphatic vasculature.

Currently, the HRA knowledge graph and API drive different 2D and 3D user interfaces in the HuBMAP, SenNet, GUDMAP, GTEx data portals and the CZ CellGuide. In line with US 6 (see Table 1), we started to develop additional lightweight web components that make it easy to access HRA data and feature HRA functionality in other websites (https://apps.humanatlas.io/us6). Plus, we are implementing diverse HRA dashboards (US 7; https://apps.humanatlas.io/dashboard) to communicate what HRA DOs exist; what experimental data is used for HRA construction (full provenance); how existing ontologies are expanded to capture healthy human terms and linkages; who is using HRA data, tools and APIs; and how representative the atlas is.

Last but not least, we will expand the interlinking of the HuBMAP Data Portal and the HRA Portal. Specifically, we will ingest new releases of the HRA into the HuBMAP UBKG so that anatomical structures, cell types and biomarkers supported in the HuBMAP Data Portal are aligned with existing ontologies and the 3D spatial reference framework. As HuBMAP teams start to compile 3D datasets, there is a need to compare existing algorithms for spatial alignment of multiple subsequent tissue sections in support of 3D tissue block reconstruction, as was done for 2D cell-segmentation methods^51,52^. 3D data are expected to considerably improve HRA quality and predictions.

Community input to HRA user stories, data, code, user interfaces and training materials is welcome and experts interested to learn more about or contribute to the HRA effort are encouraged to register for the monthly WG events online^80^.

## Methods

Human-expert-generated data and experimental tissue data are used to construct the HRA (Fig. 1). New experimental data is mapped to the HRA via (1) 3D spatial registration; (2) using suspension-based (for example, sc/snRNA-seq); or (3) spatial (for example, CODEX^83^, Cell DIVE^84^, IBEX^85,86^, Imaging Mass Cytometry^87^ and other multiplexed, antibody-based protein imaging platforms) assay types that are aligned with the HRA (Fig. 2).

### Expert-generated data

#### ASCT+B tables

ASCT+B tables (https://humanatlas.io/asctb-tables; Fig. 1a) are compiled by experts using the ASCT+B Reporter (Supplementary Fig. 4) following SOPs^88^. Note that the brain ASCT+B table is unique in that it was computationally derived using the common cell type nomenclature approach^89^, which chains together critical cell type features (for example, brain region and cortical layer), broad cell-type class and gene biomarker information into the annotation.

Starting with the sixth release of the HRA, new and revised tables list cell type parents present in CL for the about 600 cell types that currently have ASCTB-TEMP IDs (temporary ontology terms and IDs) as they do not yet exist but are systematically added into CL via the HRA effort. This makes it possible to show the complete cell typology in CellGuide (https://cellxgene.cziscience.com/cellguide) and other tools. For example, Supplementary Fig. 17 depicts a CZ CellGuide visualization for neurons (CL:0000540) showing the CL ontology typology with the ‘neuron’ cell type highlighted in green together with its parent (‘neural cell’, which is a ‘somatic cell’, which is an ‘animal cell’) and children nodes (for example, ‘GABAergic neuron’ and ‘glutamatergic neuron’). The interactive visualization is at https://cellxgene.cziscience.com/cellguide/CL_0000540.

A special focus within HuBMAP has been the development of a detailed blood vasculature ASCT+B table in support of a VCCF^90–92^ (https://humanatlas.io/vccf). Relevant data captured in the VCCF include blood vessels and their branching relationships, as well as associated cell types and biomarkers, the vessel type, anastomoses, portal systems, microvasculature, FTUs, links to 3D reference objects, vessel geometries (length and diameter) and mappings to anatomical structures the vessels supply or drain.

#### 2D and 3D reference objects

Professional medical illustrators follow SOPs^93,94^ to generate 2D reference FTU illustrations and 3D reference anatomical structures (Fig. 1b,c). Most 3D reference organs were modeled using the male and female datasets from the Visible Human Project provided by the National Library of Medicine^95^.

The ASCT+B tables in the sixth HRA release feature ontology-aligned terminology for 4,499 unique anatomical structures and 1,195 cell types. For some of these anatomical structures and cell types there exist anatomically aligned, spatially explicit reference objects. Specifically, there are 2D illustrations of 22 FTUs in 10 organs with 3,742 cells of 116 cell types plus 3D reference objects for 1,192 anatomical structures with 516 unique Uberon IDs in 65 3D reference objects (male and female, left and right organs) with 37 unique Uberon IDs. A crosswalk associates each of the 2D/3D anatomical structures and cell types with their corresponding terms in the ASCT+B tables (see SOP section)^96^.

#### Segmentation masks

Different tools are used to support manual segmentation of images by human experts (to assign each pixel in an image to an object such as a single cell, FTU or anatomical structure). Within the HRA effort, the QuPath^97^ tool is used by organ experts to generate 2D segmentation masks for FTUs and vasculature (see SOP^98,99^) and DeepCell Label (https://label.deepcell.org) is used to get 2D segmentation masks for single cells. Resulting ‘gold standard’ segmentation and annotation data (Fig. 1d) are needed to train machine-learning algorithms so that experimental datasets can be automatically segmented (Fig. 2a(v)).

#### OMAPs and AVRs

OMAPs (https://humanatlas.io/omap) are collections of antibodies designed for a particular sample preservation method and multiplexed imaging technology to allow spatial mapping of the anatomical structures and cell types present in the tissues for which they were validated^15,100^ (Fig. 1e). OMAPs are wet bench validated antibodies, which experts initially identify as candidates for their multiplexed antibody-based imaging experiments by using literature, available antibody search engines and potentially also the ASCT+B Reporter (Supplementary Fig. 4 and SOP^101^). Antibodies in OMAPs link to expert-generated HuBMAP AVRs (https://avr.hubmapconsortium.org and SOP^102^) that provide details on the characterization of individual antibodies for multiplexed antibody-based imaging assays. Antibody validation is expensive and time consuming, so these resources are designed to jump start other researchers to be successful and reduce the time and money required for multiplexed antibody-based imaging studies.

#### Cell annotation references

A large majority of single-cell data are single-cell or single-nucleus RNA-seq data. Cell-type annotation tools (Fig. 1f) such as Azimuth^14^, CellTypist^71,103^ and popular Vote (popV)^104^ are commonly used to cluster cells based on their gene expression profiles, followed by assigning those Uniform Manifold Approximation and Projection^105^ clusters to cell types based on published gene expression profiles. Supplementary Table 1 shows the number of cell types that these three tools can assign per organ (rightmost columns)—compared to the number of cell types in the ASCT+B tables and 3D reference object library (middle columns); the second column shows the number of datasets available via the HuBMAP, SenNet, GTEx and CZ CELLxGENE data portals. Note that datasets for some organs (for example, urinary bladder) do not exist.

Human expertise is required to compile crosswalks that associate cell labels assigned by these three tools to terms in CL. Mapping cell labels to CL can be partially automated; however, this is more effective if the labels researchers provide are written out rather than listed as abbreviations, as different research groups do not use standardized abbreviations for cell types. Automated mapping to CL is further hindered when the cell type is not yet present in CL, in this case, often a parent cell type is used as a placeholder until the exact cell type can be added to the ontology. For these reasons, it is desirable to construct crosswalks that use the most specific cell type supported by experimental data. Depending upon the number of active editors/curators available for adding the new cell types that single-cell RNA sequencing is discovering, prioritization of new terms and collecting supporting literature takes time. Resulting crosswalks are organ-specific and they are published as cell type annotation specific crosswalks that associate any cell type assigned by the three tools with the corresponding term in the ASCT+B tables, see examples^106^.

### Experimental data

The HuBMAP Data Portal (https://portal.hubmapconsortium.org) uses a microservices architecture (Supplementary Fig. 1) to serve data and code via a hybrid on-premises and cloud approach using federated identity management, UUIDs and full provenance for data management plus data security. Workflow and container support exist for diverse unified analysis pipelines and interactive exploration tools. This architecture makes it possible to ingest data at scale, adjust metadata formats as needed, add new algorithms and workflows as they become available and ensure production phase speed and scalability for all services. On 20 January 2024, the HuBMAP Data Portal provided open access to 2,332 datasets from 213 donors. Overall, 360 of these datasets are sc/snRNA-seq and 79 are spatial OMAP-aligned datasets.

#### Tissue collection and RUI registration

The RUI^60^ (https://apps.humanatlas.io/rui) was implemented to support the spatial registration of tissue blocks into the HRA CCF; Supplementary Fig. 6. It collects sample ID, donor metadata, plus provenance information (who registered the data and when) in the process. Subject-matter experts with knowledge of the spatial and donor data for the tissue samples use the RUI to register their tissue samples—supported by a designated HRA registration coordinator as needed, see SOP^61^. Alternatively, a more collaborative workflow is available in which the registration coordinator plays a more active role in making the registration with guidance from a subject-matter expert. These workflows are explained in two dedicated SOPs detailing how the RUI can be used^61^ and the responsibilities of the registration coordinator^107^. Next, the registration coordinator uses a location processor tool^108^ to combine tissue sample metadata with de-identified donor metadata (sex, age, body mass index, race, etc.) and publication metadata (DOI, authors, publication year, etc.). Once the samples are registered and the metadata has been enriched, the registration coordinator contacts the subject-matter expert to check accuracy and completeness. The registration coordinator then publishes the validated registration set, making it accessible through the EUI (https://apps.humanatlas.io/eui; Supplementary Fig. 7).

Tissue block registration can be streamlined and made more reproducible through the use of the ‘millitome,’ (https://humanatlas.io/millitome) a device that aids wet bench scientists to cut and register multiple tissue blocks from a single organ in a reproducible manner. This 3D-printable apparatus is designed to secure a freshly procured organ and is fitted with cutting grooves that can direct a carbon steel cutting knife for uniform slicing, see HRA’s millitome catalog (https://hubmapconsortium.github.io/hra-millitome) to access and customize organ millitomes based on donor sex, organ laterality, organ size and cutting intervals. Each millitome package contains an STL file for 3D printing the millitome’s reproducible surface geometry plus a lookup sheet correlating millitome locations with tissue sample IDs assigned by the research team. After slicing the organ using the millitome, scientists document the samples on the lookup sheet and submit this data for review by the HRA millitome facilitator. Once the package is complete, data are added to the EUI for review by scientists to verify registration accuracy in terms of tissue size, placement and orientation. SOPs detail millitome construction^109^ and usage^110^.

#### sc/snRNA-seq transcriptomic data annotation

The sc/snRNA-seq transcriptomic datasets are downloaded from four data portals using the hra-workflows-runner (https://github.com/hubmapconsortium/hra-workflows-runner). For data from HuBMAP and SenNet (each dataset comes from exactly one donor), search APIs (HuBMAP, https://search.api.hubmapconsortium.org/v3; SenNet, https://search.api.sennetconsortium.org) are used to obtain a list of dataset IDs for all existing cell-by-gene matrices in H5AD format and to download these files plus donor metadata. For GTEx, a single H5AD file is downloaded from https://gtexportal.org/home/singleCellOverviewPage. For CZ CELLxGENE, datasets are stored in collections and one collection can contain multiple datasets and donors; the workflow runner reads in an index of all healthy adult human collections compiled using the CZI Science CELLxGENE Python API (https://chanzuckerberg.github.io/cellxgene-census/python-api.html); it splits the collection into unique donor-dataset pairs; and runs all H5AD files through the three cell type annotation tools: Azimuth^14^, CellTypist^71^ and popV^104,111^ (Supplementary Table 1). Azimuth (https://azimuth.hubmapconsortium.org) serves organ-specific human adult references for ten unique organs (lung and tonsil have a revised v.2 that is used here); for Azimuth, there exists HRA crosswalks^106^ for 226 unique cell types in seven organs (3D spatial reference organs do not exist for blood, adipose tissue and bone marrow). For CellTypist (https://www.celltypist.org), there are crosswalks for 13 organs and a total of 214 unique cell types. For popV (https://github.com/YosefLab/PopV), we provide crosswalks for 22 organs and 134 unique cell types. There are 574 total cell types linked across all three tools. The workflow runner outputs four files: (1) cell summaries for all sc-transcriptomics datasets, subset by cell type annotation tool; (2) a corresponding metadata file with donor and publication information; (3) cell summaries for all sc-proteomics datasets; and (4) a corresponding metadata file with donor and publication information^112^. All four files are used during the enrichment phase to construct the atlas-level HRApop data.

#### Cell and FTU segmentation for spatial data

Antibody-based multiplexed imaging datasets, once uploaded to the HuBMAP Data Portal via the Ingest portal, are processed using a unified CWL workflow for cell and nuclei segmentation. Whole cell segmentation for CODEX datasets (https://github.com/hubmapconsortium/codex-pipeline) is conducted using Cytokit^113^ and Cell DIVE (https://github.com/hubmapconsortium/celldive-pipeline). MIBI (https://github.com/hubmapconsortium/mibi-pipeline) datasets are processed using Deepcell’s Mesmer model^114^. Resulting cell segmentations are assigned a segmentation quality score using CellSegmentationEvaluator^51^. Cell segmentation for forthcoming 3D spatial proteomics datasets is provided by 3DCellComposer^115^ in combination with trained 2D segmenters.

FTU segmentation on periodic acid–Schiff/H&E-stained histology datasets is conducted using code developed via two Kaggle competitions^77,78^. The current production pipeline includes support for FTUs in the kidney, with large intestine and spleen that will be run when histology datasets become available.

#### Cell type annotation for spatial proteomic data

After cell segmentation, spatial cell type annotation is performed using the antibody metadata for marker channels for CODEX datasets, soon expanding to MIBI and Cell DIVE. OMAPs link marker panels in the datasets to cell types in the ASCT+B tables. The SPRM package (https://github.com/hubmapconsortium/sprm) computes various statistical analyses, including mean marker expression for all cells. The Van Valen laboratory has developed a language-informed vision model, DeepCellTypes^116^ to classify cell types across tissue types and imaging technologies. This model covers 30+ cell types and will be updated as new multiplexed imaging data become available. DeepCellTypes is available at https://github.com/vanvalenlab/deepcell-types. In addition to this model, various teams have been annotating cell types with different approaches such as manual labeling with clustering or graph-based networks such as STELLAR^117^. The intestine datasets by Hickey et al.^69,117,118^ were annotated using a combination of manual and STELLAR approaches.

#### Spatial alignment for 2D multi-omics data

Spatial structural alignment of different segmentation masks, see Fig. 2a(iv) in support of multi-omics assay data analysis and/or alignment of spatial transcriptomics data to H&E imaging data can be performed using tools like STalign^119^. In STalign, segmented cellular spatial positions are rasterized into an image representation to be aligned with structurally matched H&E images. Because tissues may be rotated, stretched or otherwise warped during data collection, both affine and diffeomorphic alignments are performed. Such an alignment is achieved by optimizing an objective function that seeks to minimize the image intensity differences between a target (rasterized cell positions) and source (H&E) image subject to regularization penalties. The resulting learned transformation is applied to the original segmented cellular spatial positions to move the points into an aligned coordinate space. Such 2D spatial alignment facilitates downstream molecular and cell-type compositional comparisons within matched structures as well as integration across technologies.

#### Spatial data 3D reconstruction

Spatial alignment of multiple subsequent tissue sections in support of 3D tissue block reconstruction (Fig. 2a(vi)) has been performed using MATRICS-A^120^ for skin data. Additional tools for 3D tissue block reconstruction have been developed and include SectionAligner, 3DCellComposer^115^ and CellSegmentationEvaluator. SectionAligner takes as input a series of images of 2D tissue sections, segments each piece of tissue in each section and aligns the slices of each piece into a 3D image. 3DCellComposer uses one of various trained 2D cell segmenters (such as Mesmer), to segment each 3D image into individual cells using CellSegmentationEvaluator to automatically optimize parameter settings.

### Atlas-enriched data

#### Mesh-level collision detection

Extraction sites are post-processed via code specifically developed for efficient spatial registration using mesh surfaces^121^. To improve performance during tissue registration, the RUI uses bounding-box collision detection to determine (approximate but fast) intersections at runtime. To optimize accuracy, surface mesh collision detection is used during the enrichment phase to determine exact intersection volumes between a given RUI location and any anatomical structures it intersects with based on mesh-level colliders. The ‘3D Geometry-Based Tissue Block Annotation: Collision Detection between Tissue Blocks and Anatomical Structures’ code is available on GitHub^122^ and the API is deployed to AWS^123^.

#### HRAlit

The HRA DOs from the sixth release (for 4,499 anatomical structures, 1,295 cell types and 2,098 biomarkers) were linked to 7,103,180 publications, which are associated with 583,117 authors, 896,680 funded projects and 1,816 experimental datasets^32^. The resulting HRAlit database represents 21,704,001 records as a network with 8,694,233 nodes and 14,096,735 links. It has been mined to identify leading experts, major papers, funding trends or alignment with existing ontologies in support of systematic HRA construction and usage. All data and code are at https://github.com/cns-iu/hra-literature.

#### HRApop

HRApop provides experimental data evidence for the existence of specific cell types and mean biomarker expression values for datasets and anatomical structures for which 3D reference models exist. In the sixth release of the HRA, there are 1,192 anatomical structures of 516 types (unique Uberon IDs) for 65 organs (including male/female and left/right).

There are three criteria that experimental datasets have to meet to be used in HRApop construction: they must (1) be spatially registered using the RUI; (2) have cell type population data (for example, an H5AD file that can be annotated via CTann tools (Supplementary Table 1) or via proteomics workflows); (3) come from a data portal with quality assurance/quality control or have been published in a peer-reviewed paper.

To construct HRApop v.0.10.2, we downloaded 9,613 H5AD single-cell transcriptomics datasets from four data portals: HuBMAP, SenNet, CZ CELLxGENE and GTEx. Exactly 5,118 H5AD datasets were healthy and could be annotated using Azimuth, CellTypist or popV (Supplementary Table 1). In addition, we downloaded 74 single-cell proteomics datasets from HuBMAP published in two papers^69,120^). In sum, 553 datasets (479 sc/snRNA-seq transcriptomics and 74 spatial proteomics datasets) satisfied the three criteria and were used for HRApop construction.

The resulting HRApop v.0.10.2 was validated and optimized by making predictions for datasets for which both an RUI-registered extraction site and a cell type population exist. It was then used to predict cell type annotation or spatial origin for 2,004 HuBMAP, 166 SenNet and 4,789 CZ CELLxGENE datasets for which this information was missing.

#### VCCF distances and Vitessce visualizations in 2D

In support of constructing a VCCF^90–92^, code that measures and graphs the distance of different cell types to blood vessel cell types in 2D and 3D (see SOP^124^) has been developed. Distance plots can be overlaid on the tissue section using Vitessce^62,63^ for 2D data and explored in 2D and 3D using custom code^120,125^; Figs. 2a(viii) and 3a provide examples. A new tool, Cell Distance Explorer, is also made available to visualize cell-to-cell graphs and distance distributions for both 2D and 3D datasets (https://apps.humanatlas.io/cde).

#### Hierarchical community analysis of cell types

Hierarchical community analysis of cell types makes it possible to automatically detect multilevel FTUs^69^. The approach uses the single-cell labels and *x*, *y* coordinates from spatial datasets. For the preview example featured in this paper (Fig. 3b), the dataset is a CODEX multiplexed imaging^83,118,126^ dataset of the healthy human intestine^69^. The original multiplexed imaging data was segmented, normalized and clustered using z-normalization of the antibody markers used with Leiden unsupervised clustering^100^. Cell types were propagated to additional samples using the deep-learning algorithm (STELLAR) for cell type label transfer in spatial single-cell data^117^.

Once cell type labels were assigned, cell neighborhoods were calculated by clustering nearest neighbor (*n* = 10) vectors surrounding each cell. A similar approach was taken to identify larger structures (termed communities^69^) using neighborhoods as the labels and taking a larger window for the nearest neighbors (*n* = 100). Similarly, to identify major tissue units, community labels were used and an even larger window for nearest neighbors (*n* = 300) before clustering of the vectors. Once all tissue structures were identified, the connections in terms of primary components from various levels of tissue structures can be connected and visualized via a network graph. Currently, each node is organized per level and connected to the next spatial layer (for example, cell type to neighborhood, neighborhood to community). This code is deposited on GitHub^67^.

### Atlas validation

Each DO in the HRA is validated either by human expert review or using algorithmic means. HRA DO data formats depending on the type: ASCT+B is in CSV format, 3D Reference Organs in GLB format, 2D FTUs in scalable vector graphics, etc. When these data are normalized to LinkML format, the source data are processed and structural errors in the raw data are identified. Once in a normalized form, LinkML is used to validate the structure of the transformed data, including ensuring that data types and URLs fall within acceptable parameters. This step catches basic errors, including malformed URLs, missing data and incorrect data types that can be a problem downstream. Beyond this, certain DO types go through more advanced semantic checks to be sure that ontology terms used actually exist and that assertions from the DO also appear in trusted ontologies like Uberon and Cell Ontology. Validation of the ASCT+B tables is most rigorous and involves detailed review and reporting from the EBI team^127^. While these tables are being authored, new/updated terms and relationships are published in the latest ontology versions available in Ubergraph (Uberon 2024-01-18 and CL 2024-01-05 for the sixth HRA) and weekly reports are generated at https://hubmapconsortium.github.io/ccf-validation-tools/ to aid table authors in getting the highest quality data for the HRA.

### Flexible hybrid cloud microservices architecture

#### Hybrid cloud

The IEC developed a hybrid cloud infrastructure that leverages the unique strengths of both on-premises and public cloud resources—each colocating robust and scalable storage with robust and scalable computing—providing the flexibility to proactively adapt to evolving technologies and respond to the needs of the HuBMAP Consortium and the broader atlasing community. As a key piece of this strategy, the HIVE IEC ingested, processed and archived HuBMAP data at PSC. This approach provides flexible access, as the primary copy of HuBMAP data can be stored on-premises at a low cost, but then made available on any public or local resource without incurring substantial industry standard data egress charges, as well as free, low-friction access, as researchers can run basic analyses without having to create a public cloud account or larger analyses by accessing the full HuBMAP data repository colocated with PSC’s national supercomputing infrastructure made available without charge to the research community.

#### Microservices architecture

The HuBMAP microservices architecture (Supplementary Fig. 1) is built via agile development practices based on user-centered design, with microservices that communicate using REST APIs^128^ via Docker orchestration on AWS and on-premise resources. Each microservice is focused to serve specific functionality. Services are packaged into individual Docker containers. This orchestration of Docker containers is routinely built and rebuilt in development, test and production instances which allows for independent operation and monitoring. This microservices architecture supports the plug-and-play of a continuously evolving set of algorithms required for experimental data ingestion, annotation, segmentation, search, filter and visualization, as well as for atlas construction and usage. Supplementary Fig. 1 shows the resource, API and application layers with exemplary modules (the Supplementary Information website shows an interactive version that lets users click on any module to access details). The core service that others are dependent on is the Entity API, backed by a Neo4j graph database, which provides the storage (creation, retrieval, update and deletion) of all provenance and metadata information associated with HuBMAP data. The Search API allows for search of all provenance and metadata via the AWS hosted OpenSearch search engine, which holds a copy of all information maintained by the Entity API. The HuBMAP authentication and authorization model makes use of the Globus Auth service (https://globus.org) with login services in compliance with the OAuth2 standard (https://oauth.net/2), which provides user tokens that are passed among the services where they can be centrally validated and provide user authorization with linkage to defined groups via the Globus Groups service. The remaining services provide application specific functionality for support of data ingest and management (Ingest API) and unique entity tracking and ID generation (UUID API).

#### HRA cloud infrastructure

HRA applications, including the HRA Portal, HRA Knowledge Graph, EUI and RUI are all deployed to the web and hosted via AWS or GitHub pages. For applications requiring server-side logic, Docker containers are created, tested and built automatically with continuous integration/continuous deployment via GitHub Actions, published to Amazon Elastic Container Registry and then deployed via AWS AppRunner or Amazon Elastic Container Service. For applications which are served primarily as static files, they are tested and built automatically with CI/CD via GitHub Actions and then copied to Amazon S3 for serving or pushed to a branch for GitHub pages deployment. Except for GitHub Pages, both static and server driven applications have Amazon CloudFront act as the front-end, providing a service mesh that supports serving web requests, tracking usage, proxying requests to services running in AWS AppRunner or Elastic Container Service and caching frequently used files and responses. While AWS are used extensively in the HRA cloud infrastructure, the technology is well suited to be adapted to other platforms.

### Reporting summary

Further information on research design is available in the Nature Portfolio Reporting Summary linked to this article.

## Online content

Any methods, additional references, Nature Portfolio reporting summaries, source data, extended data, supplementary information, acknowledgements, peer review information; details of author contributions and competing interests; and statements of data and code availability are available at 10.1038/s41592-024-02563-5.

## Supplementary information

Supplementary Information Supplementary Figs. 1–17 and Tables 1–3.

Reporting Summary

## Data Availability

All HuBMAP data are available via the HuBMAP Data Portal at https://portal.hubmapconsortium.org. Azimuth references can be accessed at https://azimuth.hubmapconsortium.org. HRA data and code are available at the HRA Portal (https://humanatlas.io). HuBMAP and HRA primary and secondary data repositories are listed in Supplementary Table 2 and HRA code repositories are in Supplementary Table 3.

## Code availability

Code is available on three different GitHub organizations: (1) https://github.com/hubmapconsortium is the HuBMAP organization where HRA started; (2) https://github.com/cns-iu is the organization owned by the Cyberinfrastructure for Network Science Center at Indiana University and initial experimental HRA code starts here; and (3) https://github.com/x-atlas-consortia was created recently to host cross-consortia code, including hra-kg, hra-pop, hra-apps and hra-api. Supporting information is at https://cns-iu.github.io/hra-construction-usage-supporting-information.
