# Supplementary material for: Human BioMolecular Atlas Program (HuBMAP): 3D Human Reference Atlas construction and usage
Source: Nat Methods. 2025 Mar 13;22(4):845–60. doi: 10.1038/s41592-024-02563-5 (PMC11978508; doi:10.1038/s41592-024-02563-5)
Supplement: Supplementary file 1 — Supplementary Figs. 1–17 and Tables 1–3. [file 41592_2024_2563_MOESM1_ESM.pdf]

nature methods

Supplementary information https://doi.org/10.1038/s41592-024-02563-5

Human BioMolecular Atlas Program (HuBMAP): 3D Human Reference Atlas construction and usage

In the format provided by the authors and unedited

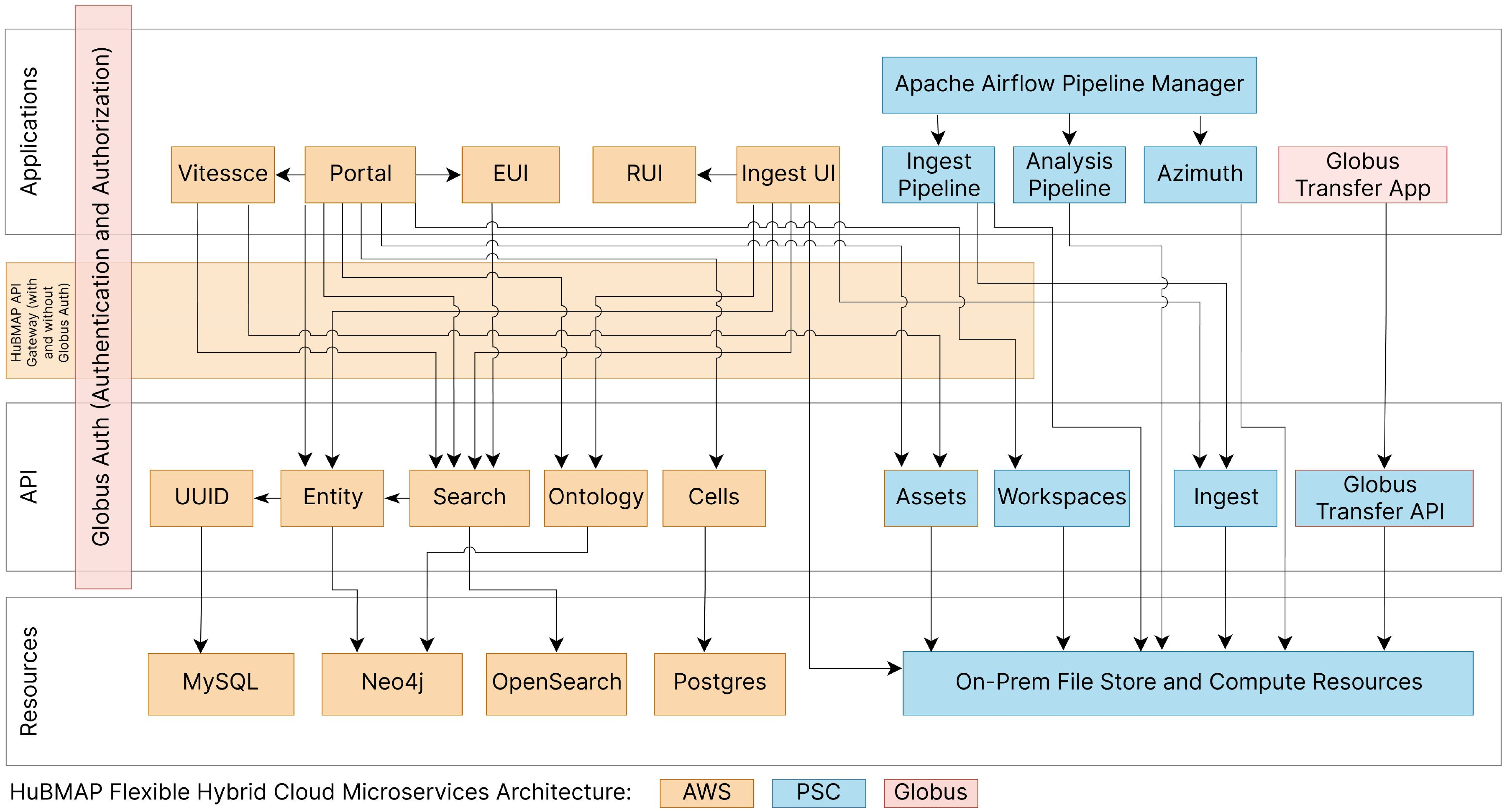

Supplemental Figure 1. Hybrid Cloud Microservices System Architecture

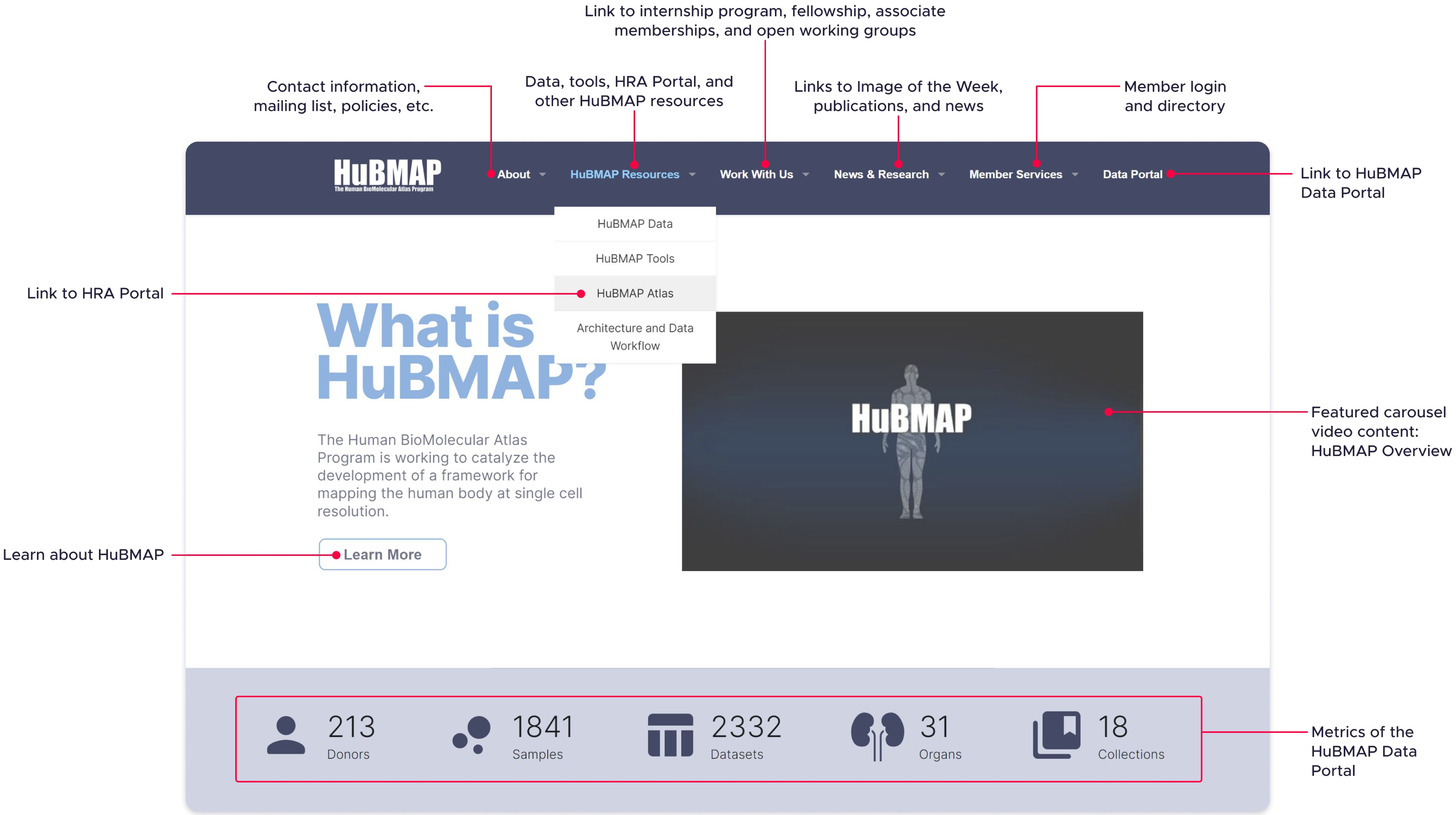

Supplemental Figure 2. HuBMAP Consortium Website

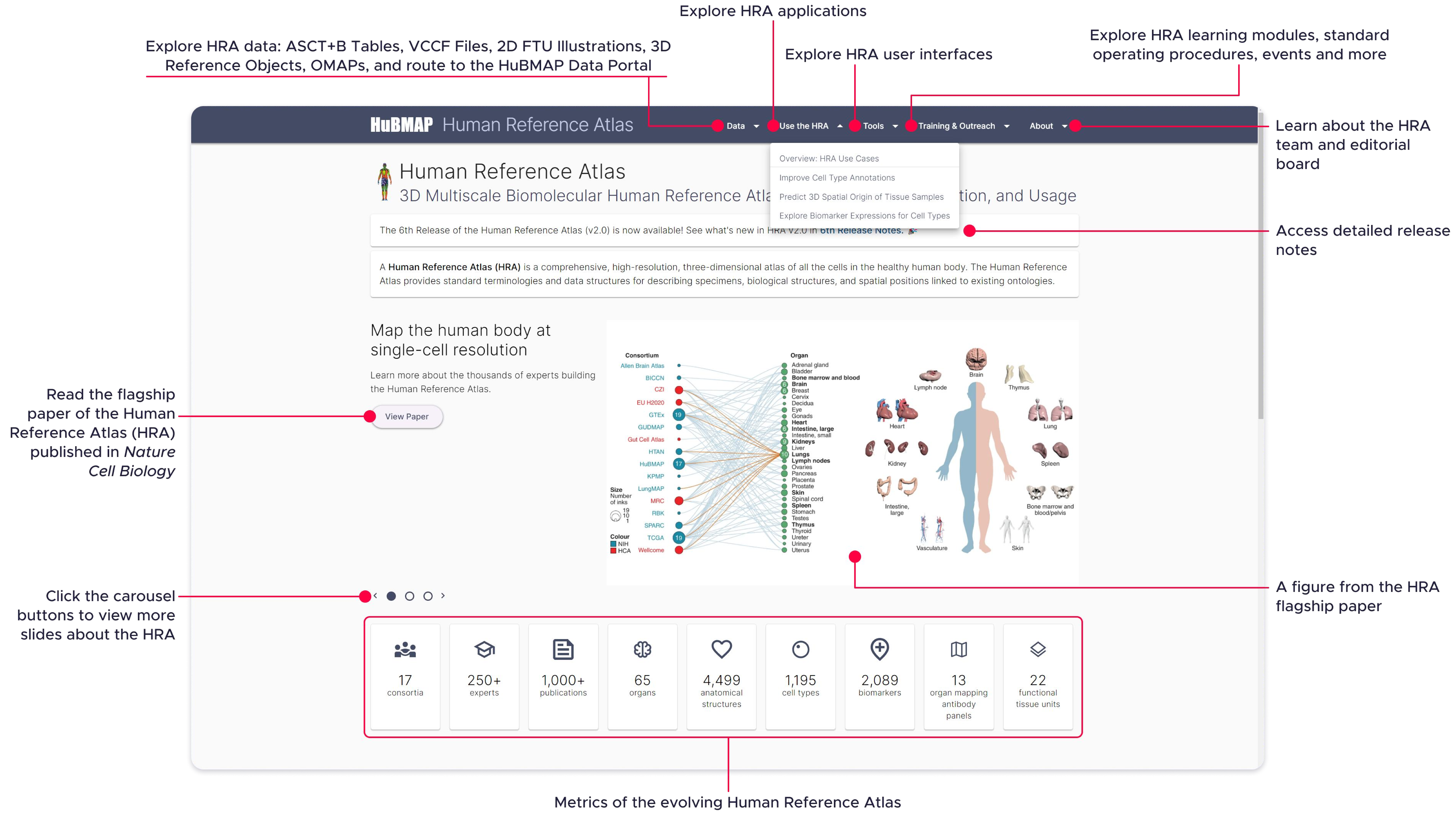

Supplemental Figure 3. Human Reference Atlas Portal

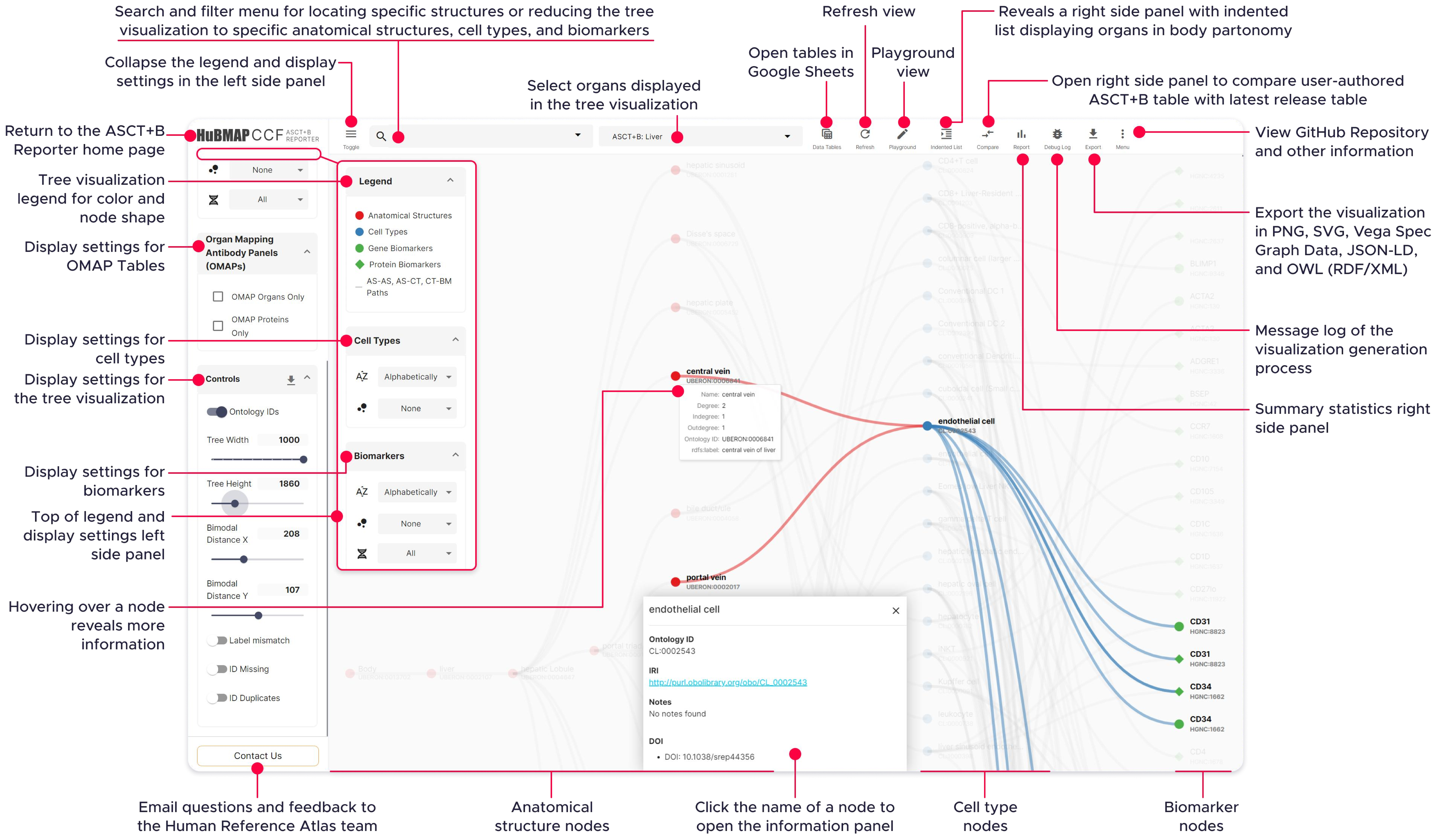

Supplemental Figure 4: ASCT+B Reporter User Interface

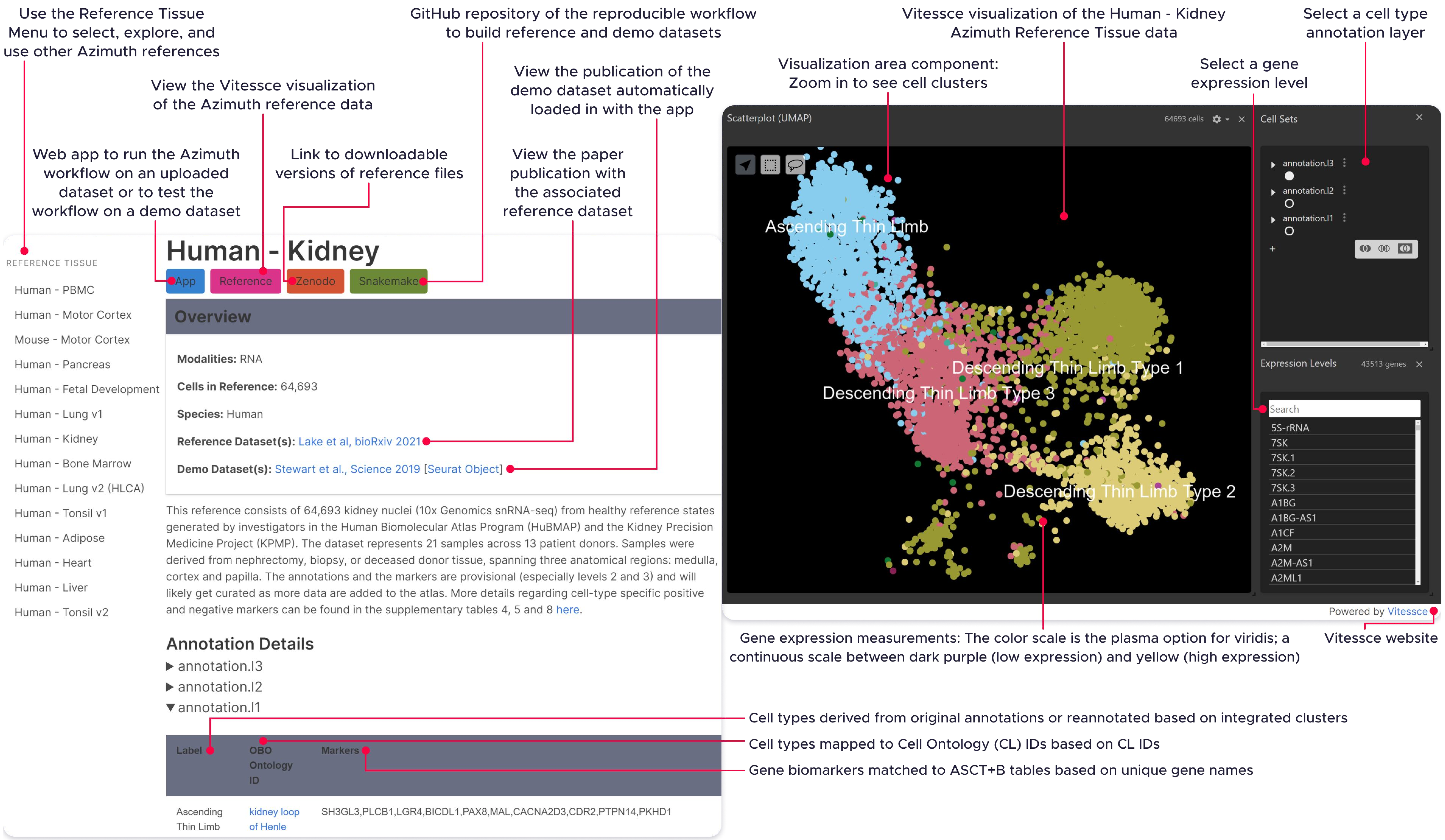

Supplemental Figure 5. Azimuth Portal and Reference Explorer User Interface

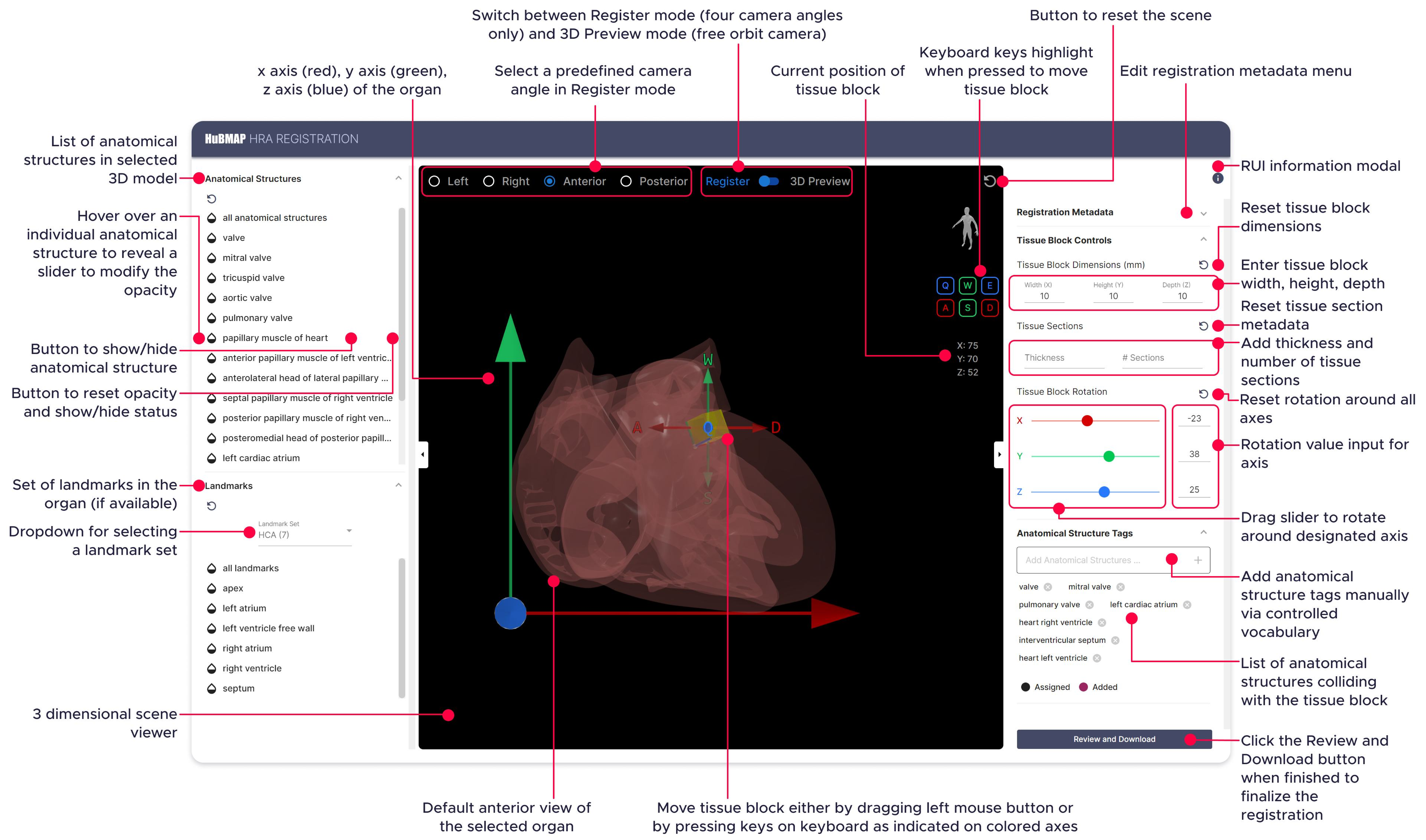

Supplemental Figure 6. Registration User Interface (RUI)

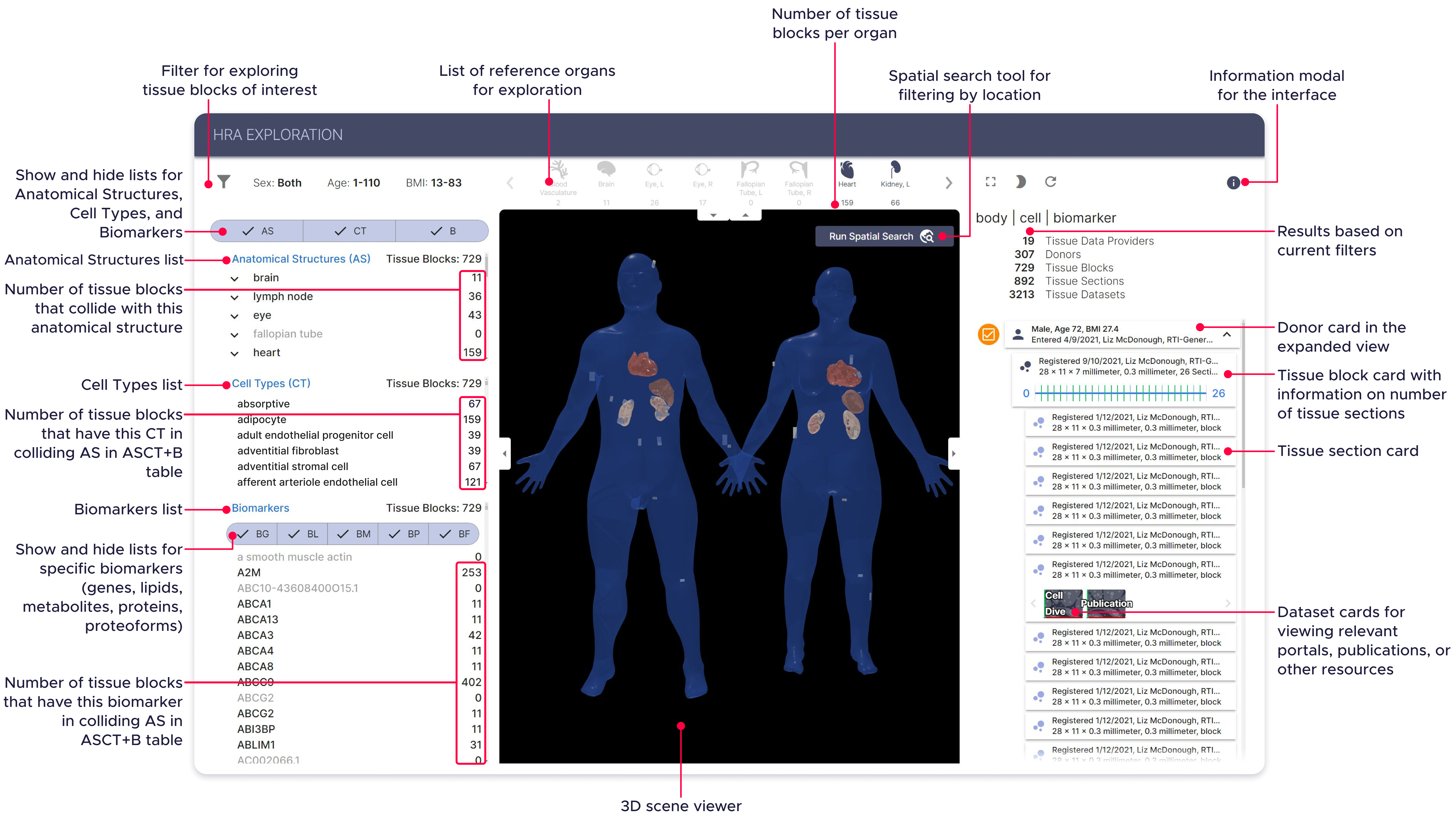

Supplemental Figure 7: Exploration User Interface (EUI)

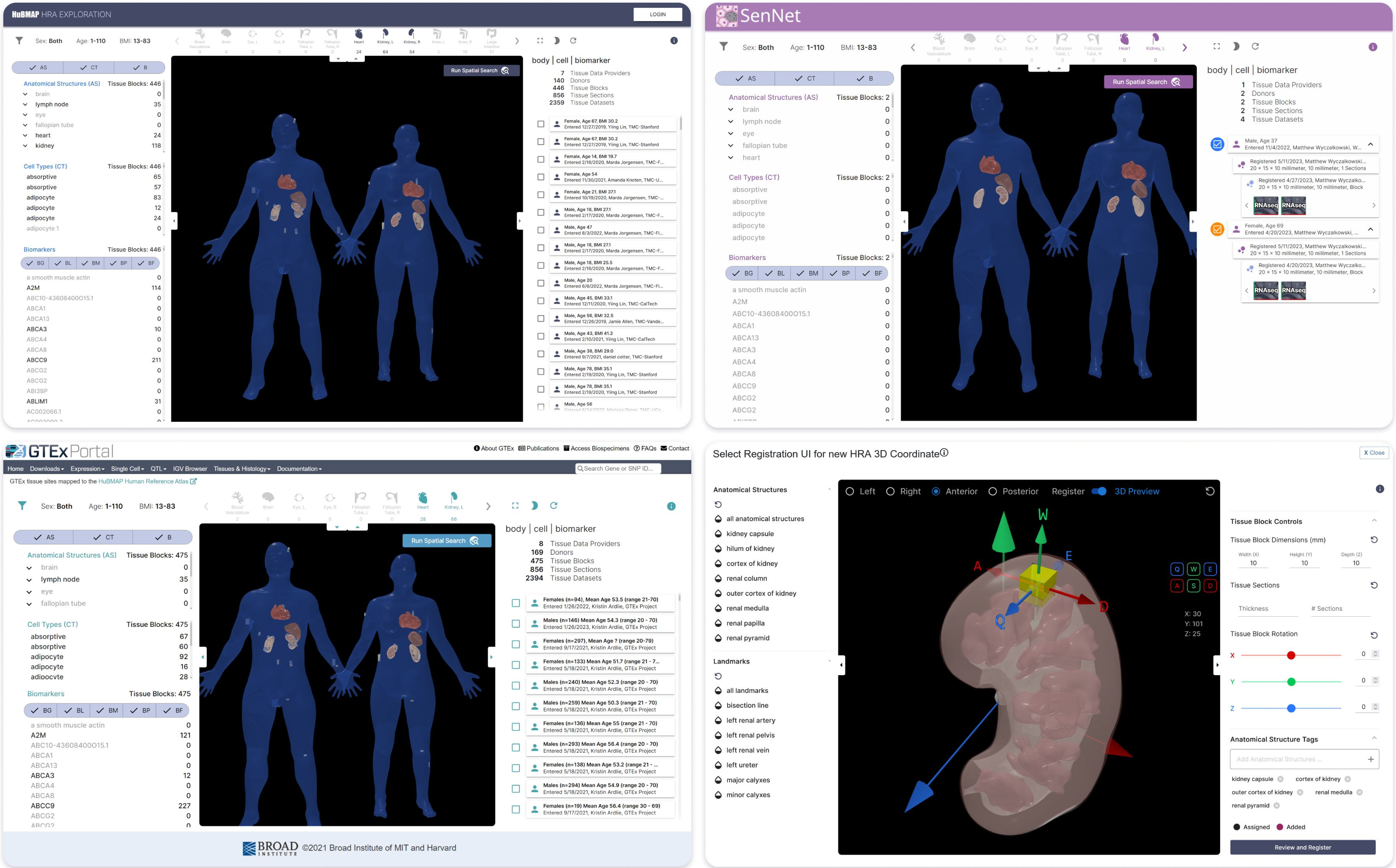

Supplemental Figure 8: Customized, Branded Deployment of EUI in HuBMAP, SenNet, GTEx, and RUI in GUDMAP

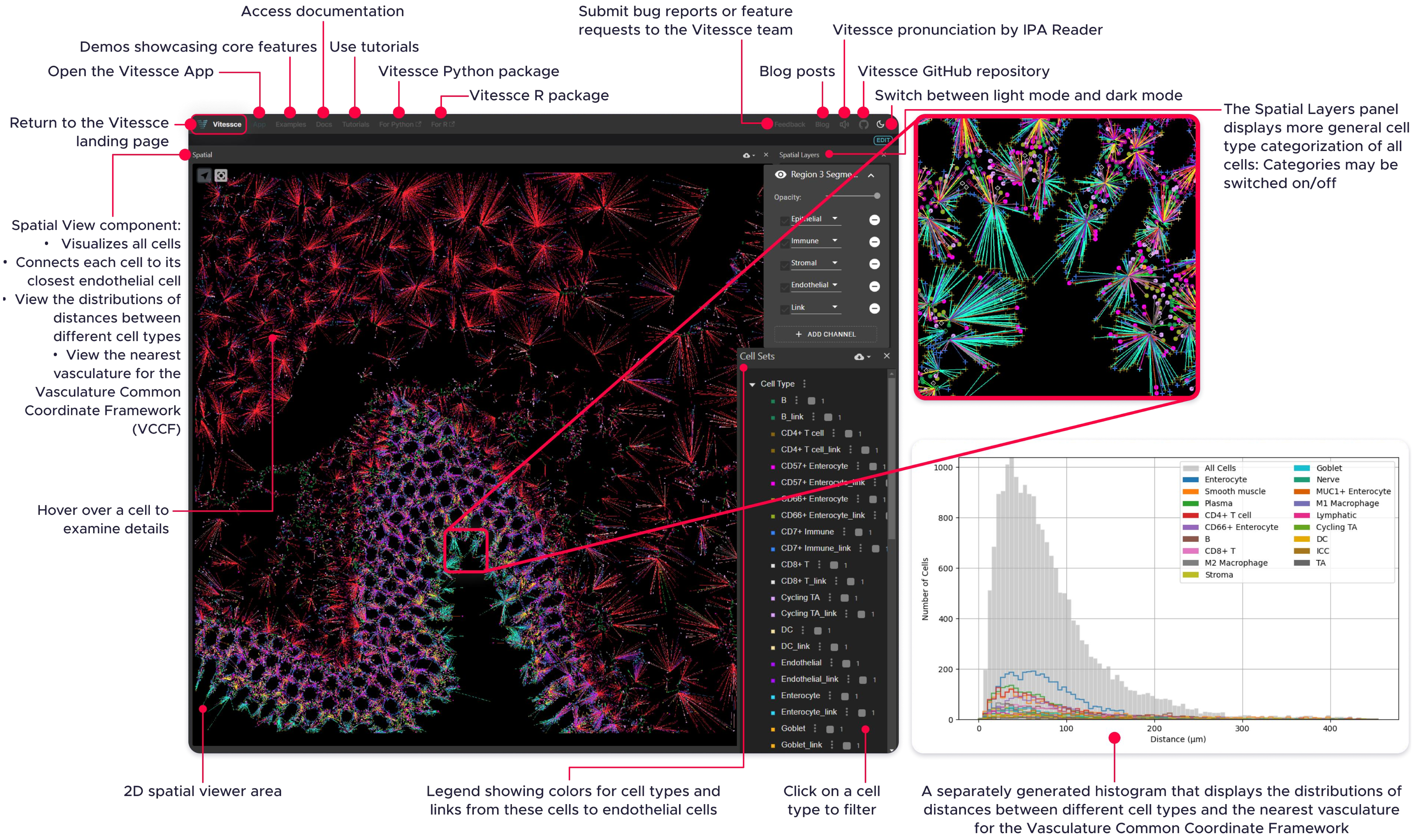

Supplemental Figure 9: Vasculature Common Coordinate Framework Distance Visualizations

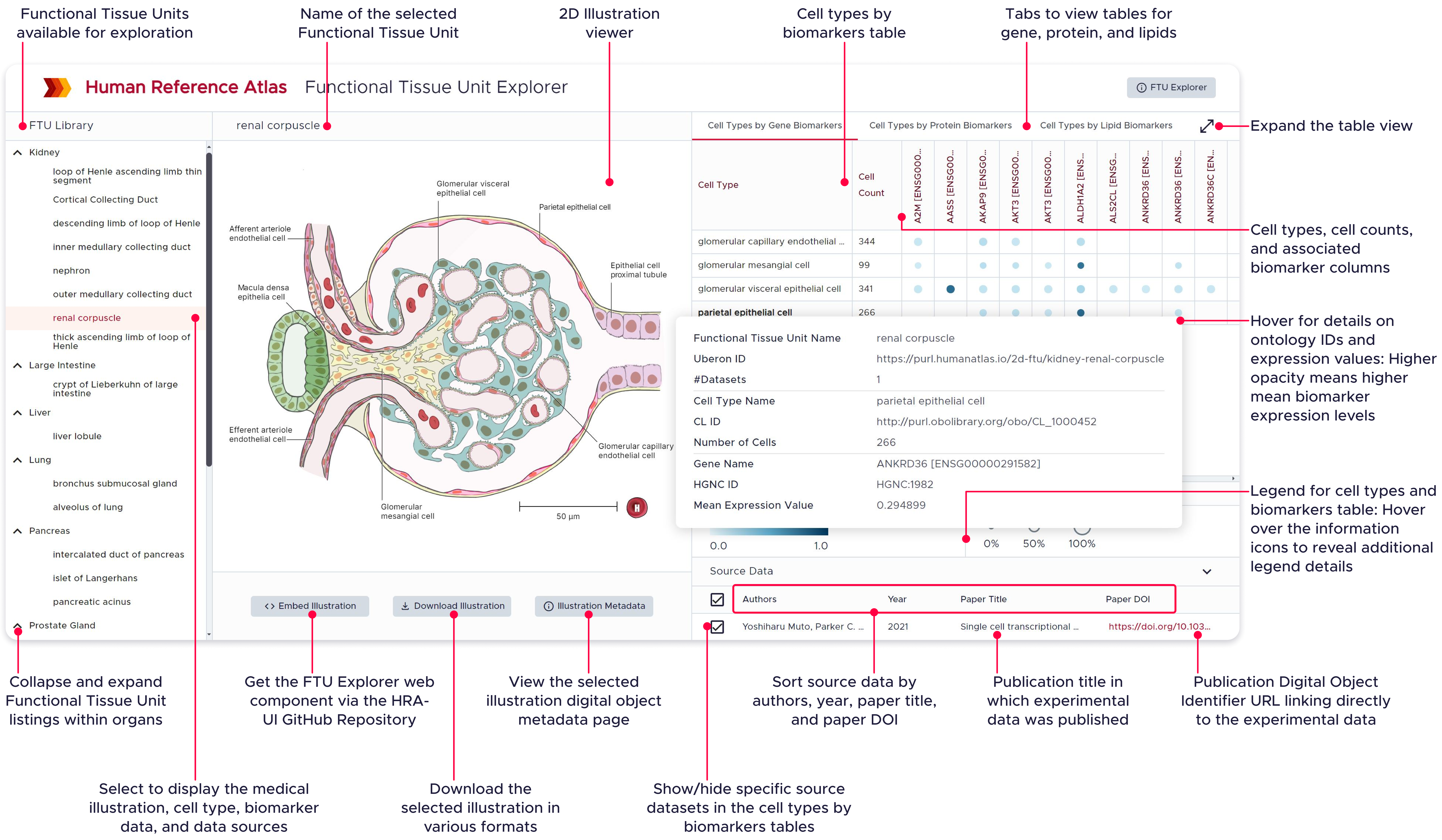

Supplemental Figure 10: Interactive FTU Explorer

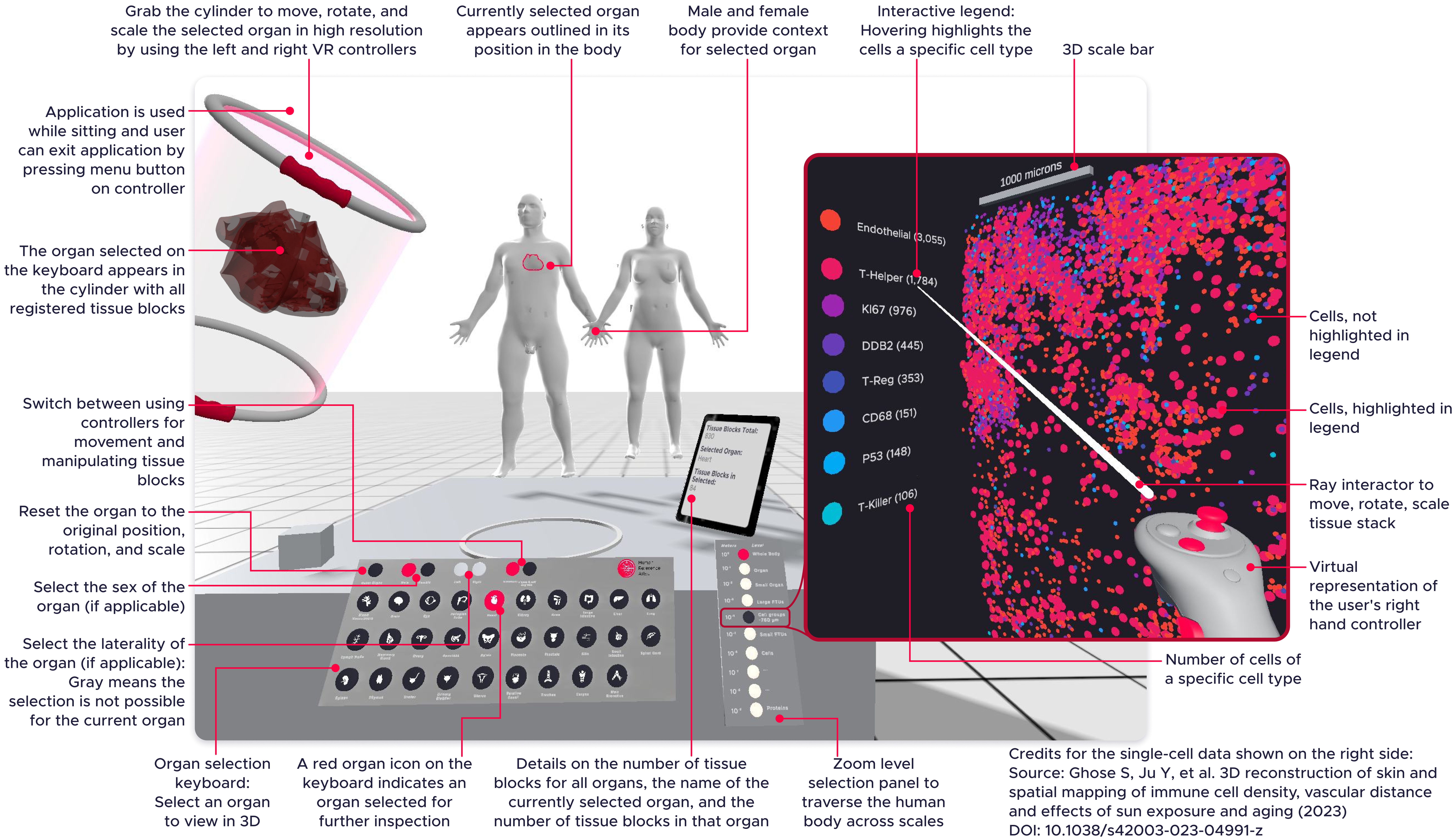

Supplemental Figure 11: HRA Organ Gallery

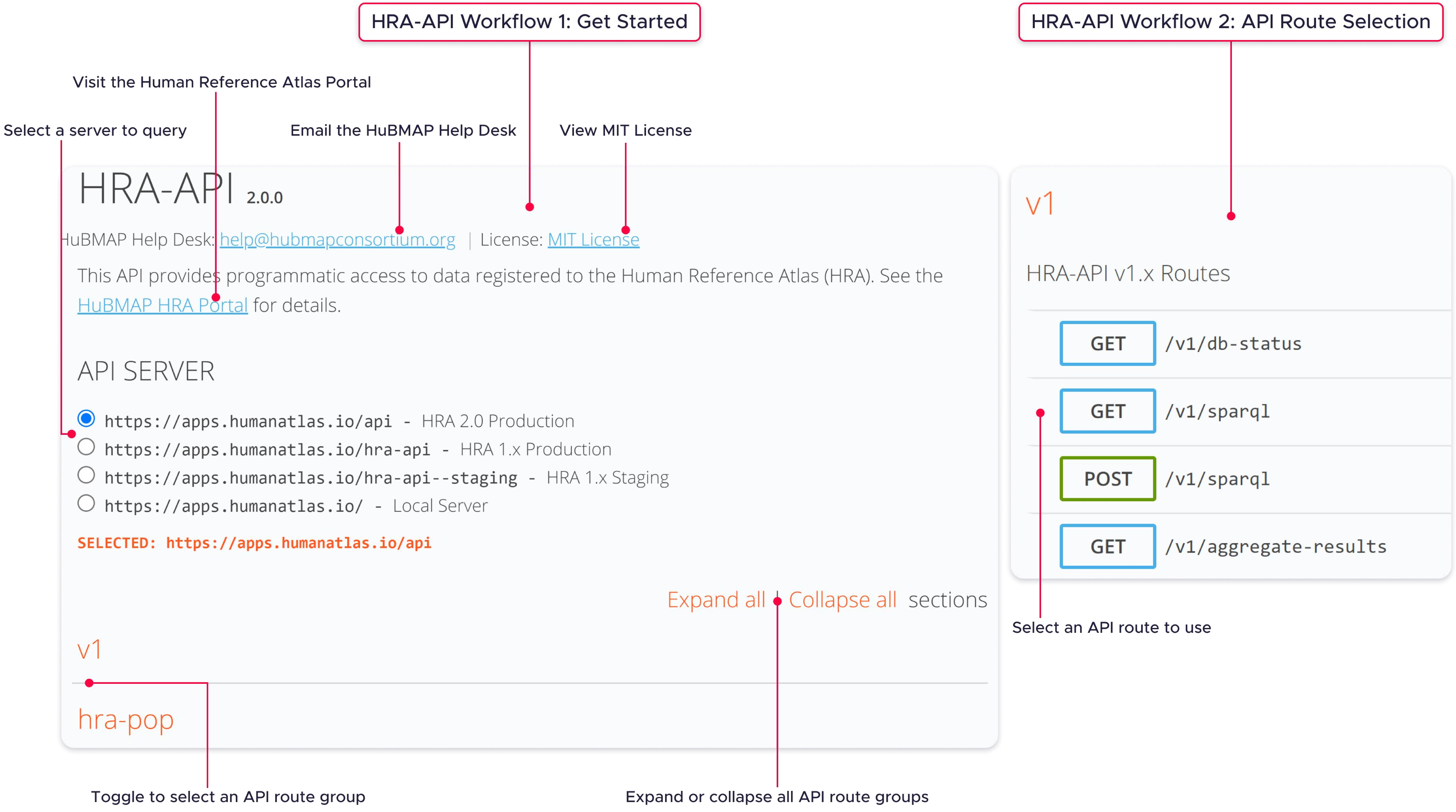

Supplemental Figure 12: Human Reference Atlas Application Programming Interface: Get Started and API Route Selection

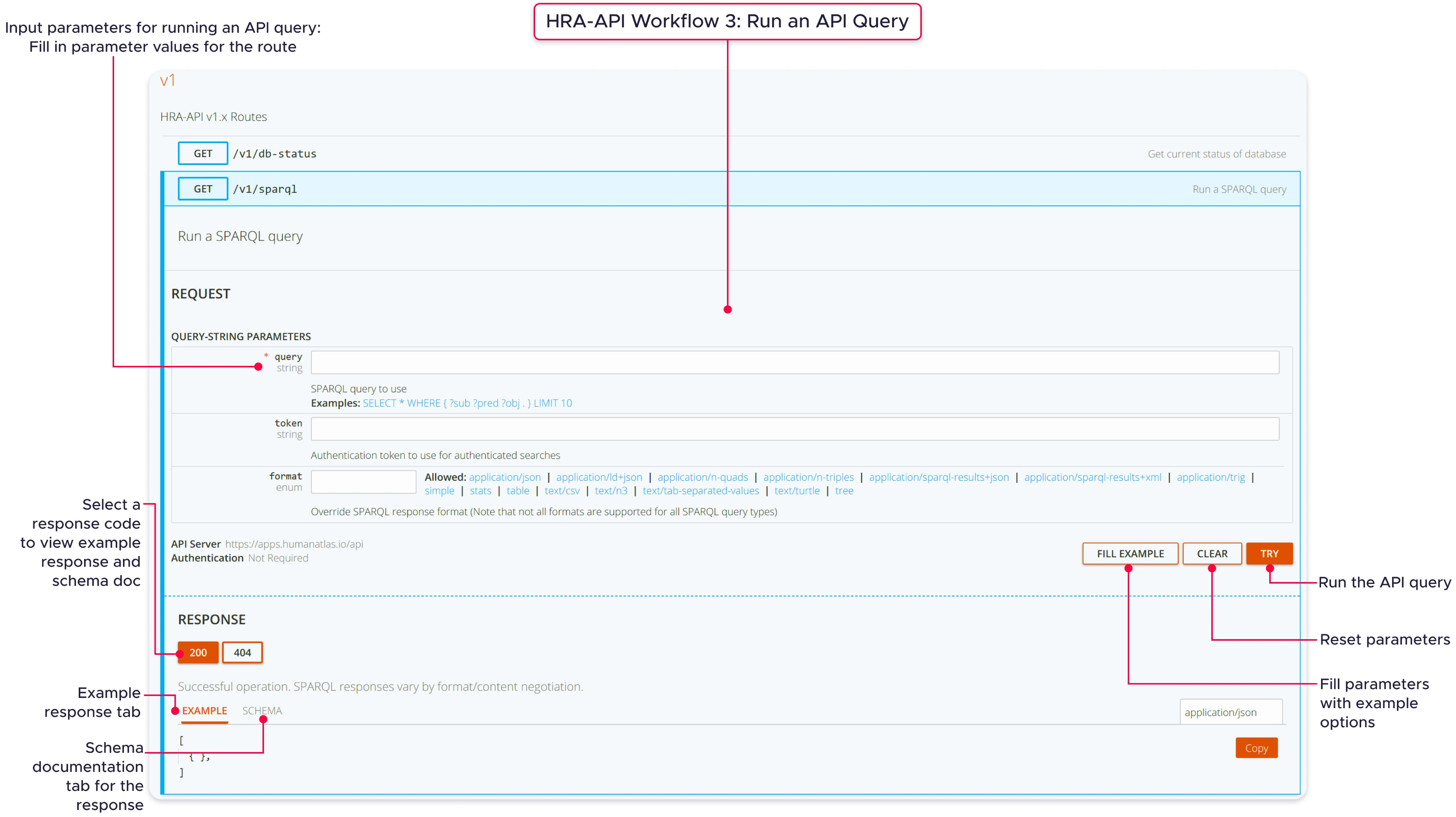

Supplemental Figure 13: Human Reference Atlas Application Programming Interface: Run an API Query

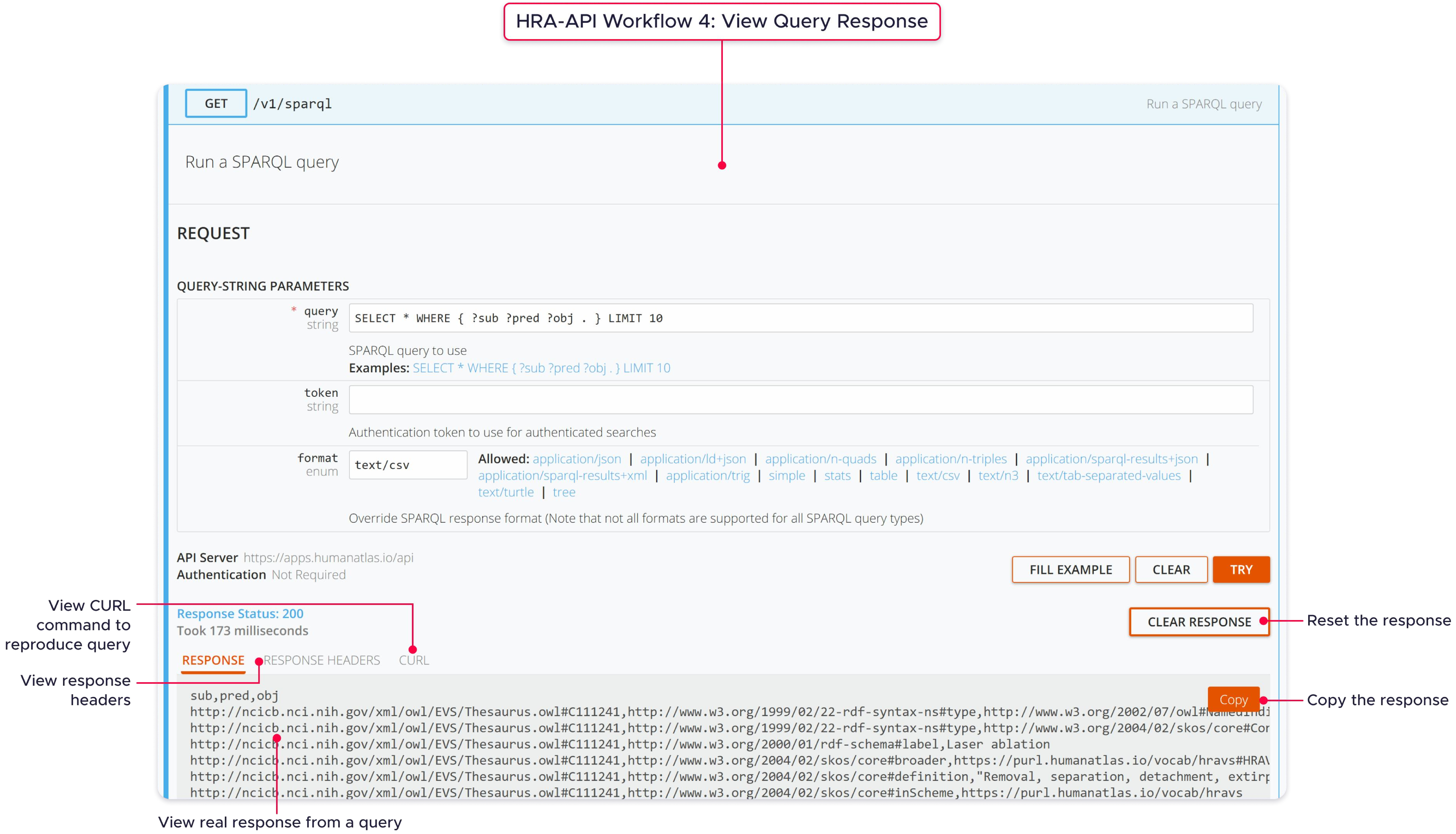

Supplemental Figure 14: Human Reference Atlas Application Programming Interface: View Query Response

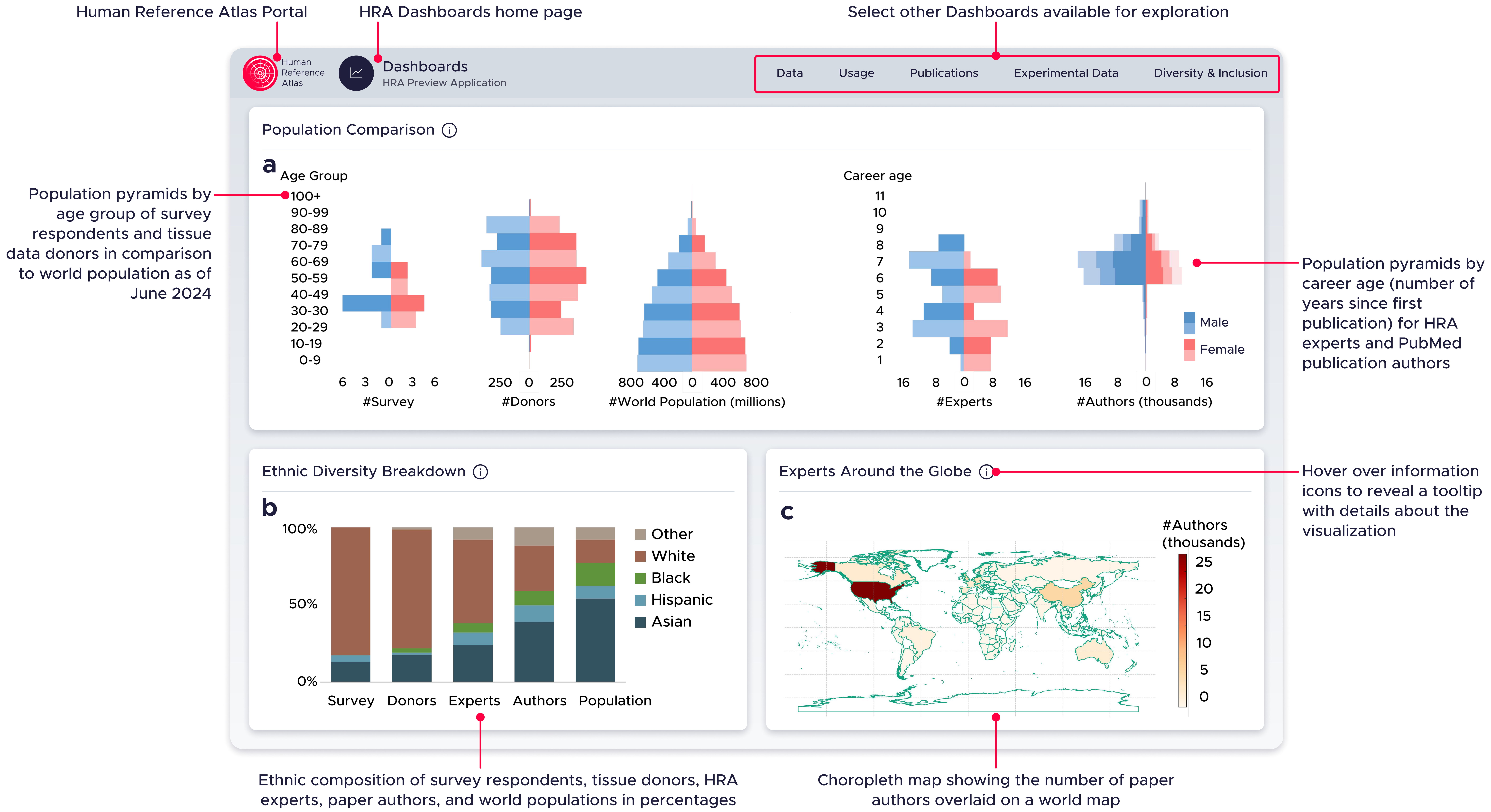

Supplemental Figure 15. HRA Equity Dashboard

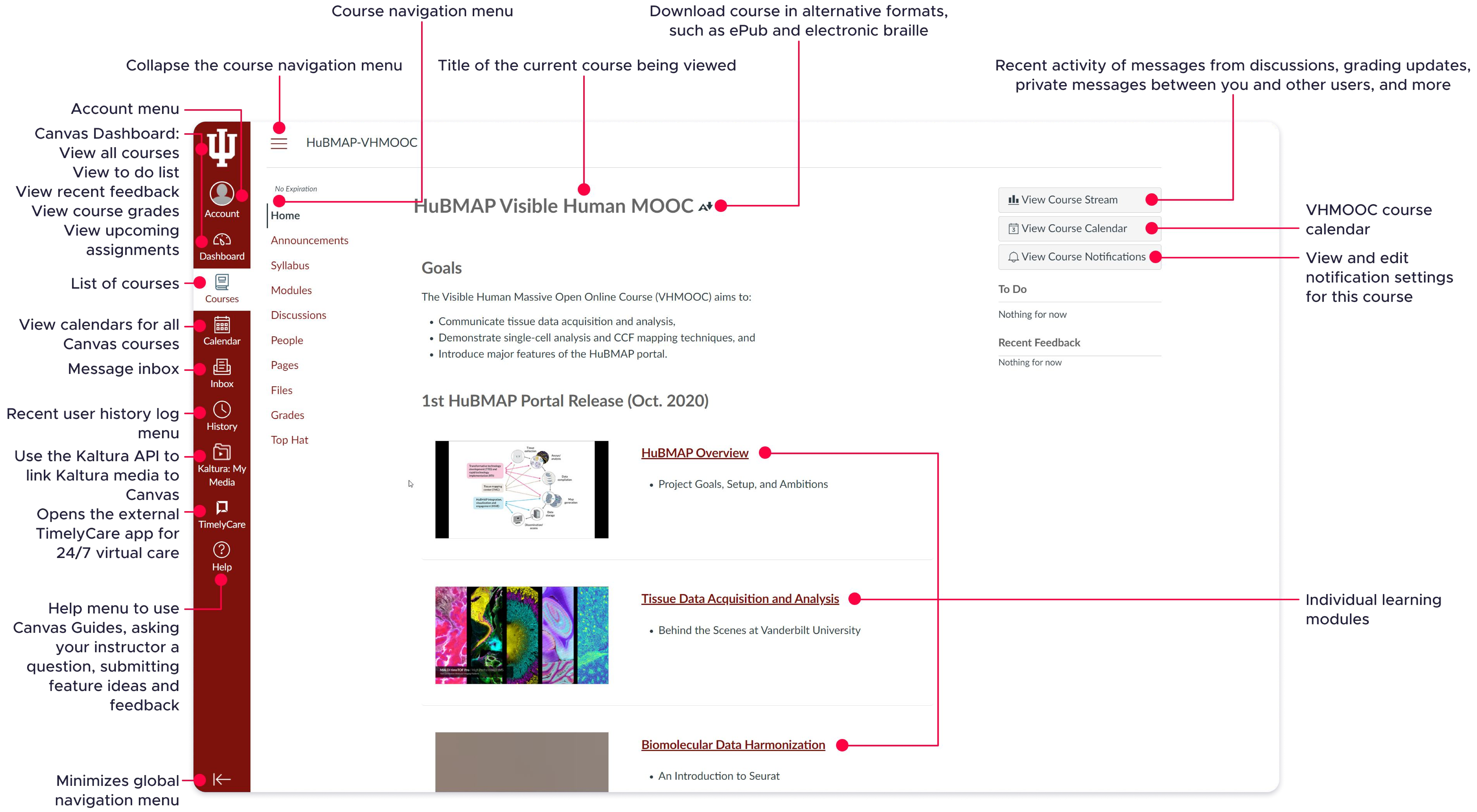

Supplemental Figure 16: Visible Human Massive Open Online Course (MOOC)

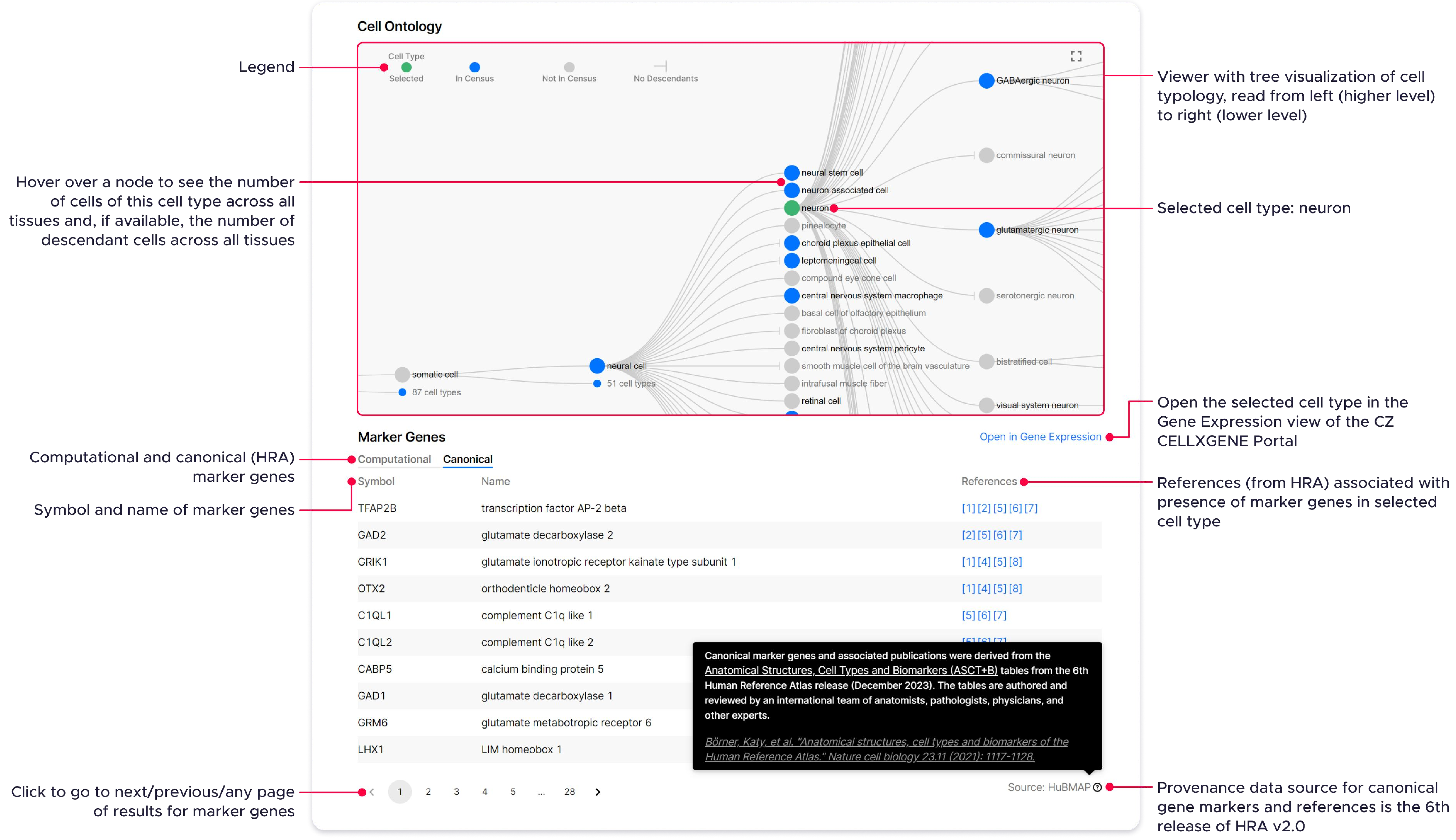

Supplemental Figure 17: CZ CellGuide Visualization With 'Canonical' Marker Genes And 'References' From The HRA

Supplemental Tables

Supplemental Table 1: Experimental Data, HRA Data and Cell Type Annotation by Organ. Number of sc/snRNA-seq datasets per organ vs. number of 3D reference organs plus anatomical structure and unique cell types in ASCT+B tables vs. number of cell types that different cell type annotation tools can assign across all annotation levels.

		ASCT+B and 3D Reference Organs			Cell Type Annotation Tools		
**Organ**	**Datasets with H5AD File**	**#AS in 3D (Male+Female)**	**#AS**	**#CT**	**Azimuth**	**CellTypist**	**popV**
blood	2,501	none	1	29	41	26	18
blood vasculature	3	244	1,054	10	0	0	14
bone marrow	131	none	1	47	43	39	14
brain*	656	210	188	625	0	0	0
brain > motor cortex*	Part of 656	4**	1	139	20	0	0
brain > hippocampus*	Part of 656	12**	4	45	0	20	0
breast/mammary gland	1	18	3	10	0	0	14
eye	192	96	39	55	0	0	27
heart	254	34	52	28	25	49	6
kidney	207	116	61	70	58	34	0
large intestine	140	22	54	58	0	0	16
liver	70	46	17	30	25	35	12
lung	531	141	54	74	78	71	36
lymph node***	16	16	34	45	0	28	22
pancreas	14	11	31	30	11	9	14
prostate gland	34	18	13	19	0	0	13
skin of body	53	2	15	35	0	23	21
small intestine	177	23	39	34	0	0	16
spleen	39	12	37	59	0	32	22
thymus	47	6	18	50	0	0	21
trachea	29	8	20	17	0	0	18
urinary bladder	0	15	16	15	0	0	14
uterus	23	10	61	18	0	0	13
**Total (sum, not unique)**	**5,118**	**1,064**	**1,810**	**1,542**	**301**	**366**	**331**

* Azimuth and CellTypist focus on the motor cortex and hippocampus, respectively. The 3D Reference Object and ASCT+B Table of the brain in the HRA focus on the entire organ.
** Primary motor cortex is part of the precentral gyrus and hippocampus has three (head, body, tail) 3D reference objects in each hemisphere in male and female.
*** The 3D lymph node is generic and was placed in the mesenteric region as all HuBMAP data is from that region.

Supplemental Table 2: Primary and Secondary HRA Data Repositories. The rightmost column shows data used in: HuBMAP Data Portal (H), HRA Portal (A), demonstration previews (P), and external code (E).

Object Type	Primary data repository	Data mirrors	Type
UBKG	https://ubkg.docs.xconsortia.org		H
HuBMAP Ontology API	https://smart-api.info/ui/d10ff85265d8b749fbe3ad7b51d0bf0a		H
CCF.OWL v2.3.0	https://lod.humanatlas.io/graph/ccf	https://www.ebi.ac.uk/ols/ontologies/ccf<br>https://bioportal.bioontology.org/ontologies/CCF<br>https://ontobee.org/ontology/CCFO	A
HRA KG	https://lod.humanatlas.io		A
HRA ASCT+B Tables	https://humanatlas.io/asctb-tables	https://commons.datacite.org/repositories/8orcvek<br>https://datasetsearch.research.google.com/search?query=HuBMAP	A
HRA OMAPs	https://humanatlas.io/omap	https://cdn.humanatlas.io/hra-releases	A
AVRs	https://avr.hubmapconsortium.org	https://cdn.humanatlas.io/hra-releases	H
HRA 2D references	https://humanatlas.io/2d-ftu-illustrations	https://cdn.humanatlas.io/hra-releases	A
HRA 3D references	https://humanatlas.io/3d-reference-library	https://cdn.humanatlas.io/hra-releases<br>NIH3D: https://3d.nih.gov/collections/hra<br>EMBL: https://www.ebi.ac.uk/ols4/ontologies/ccf, e.g., brain is at http://purl.obolibrary.org/obo/UBERON_0000955	A
HRA VCCF	https://humanatlas.io/vccf	https://cdn.humanatlas.io/hra-releases	A
Azimuth	https://azimuth.hubmapconsortium.org		H
CellTypist	https://www.celltypist.org/models	https://pypi.org/project/celltypist/	E
popV	https://github.com/YosefLab/PopV	https://zenodo.org/records/7580707	E
FTU Segmentation training data	HuBMAP Collection doi:10.35079/hbm925.sgxl.596	https://zenodo.org/records/7729610<br>https://zenodo.org/records/7545745	H
"Hierarchical cell type populations within FTUs" data	https://portal.hubmapconsortium.org/browse/publication/77ab35880329b5932380104aa58795a4	https://doi.org/10.5061/dryad.pk0p2ngrf<br>https://doi.org/10.5061/dryad.76hdr7t1p (Part 1)<br>https://doi.org/10.5061/dryad.gmsbcc2sq (Part 2)	P
"Perivascular immune cells in lung" data	https://drive.google.com/drive/folders/1SPbN6_0C-mVWbfE5r2OTVZLMxBjiEKnI?usp=sharing		P

Supplemental Table 3: HRA Code Repositories. The rightmost column shows data used in: HuBMAP Data Portal (H), HRA Portal (A), demonstration previews (P), and external code (E).

Code Type and Name	GitHub Repository	Type
**Data processing**		
QuPath Manual Segmentation Tool (0.5.1)	https://qupath.github.io	E
Azimuth (0.4.6)	https://github.com/hubmapconsortium/azimuth-annotate	H
CellTypist (1.6)	https://github.com/Teichlab/celltypist	E
popV (0.9)	https://github.com/YosefLab/PopV	E
Van Valen Tools	https://github.com/vanvalenlab?q=deep	E
Van Valen's Cell Type Annotation (HuBMAP internal)	https://github.com/vanvalenlab/deepcell-types	E
HuBMAP sc/snRNA-seq and CODEX pipelines	https://github.com/hubmapconsortium/salmon-rnaseq<br>https://github.com/hubmapconsortium/codex-pipeline	H
3DCellComposer (1.2)	https://github.com/murphygroup/3DCellComposer	P
CytoSpatio (1.0.0)	https://github.com/murphygroup/CytoSpatio	P
PanelOptimizer	https://github.com/murphygroup/CODEXPanelOptimization	P
CellSegmentationEvaluator (1.5)	https://github.com/murphygroup/CellSegmentationEvaluator	H
FTU segmentation via Kaggle #1-2 Competitions	https://github.com/hubmapconsortium/pas-ftu-segmentation-pipeline<br>https://github.com/cns-iu/hra-multiftu-segmentation-pipeline	H
Cell Neighborhood Analysis	https://github.com/HickeyLab/Hierarchical-Tissue-Unit-Annotation	P
STELLAR	https://github.com/snap-stanford/stellar	P
STalign (1.0.1)	https://github.com/JEFworks-Lab/STalign	P
HRA Dataset Graphs Library (1.0)	https://github.com/hubmapconsortium/hra-rui-locations-processor	A
HRA Digital Object Processor	https://github.com/hubmapconsortium/hra-do-processor	A
HRA Workflows	https://github.com/hubmapconsortium/hra-workflows	A
HRA Workflows Runner	https://github.com/hubmapconsortium/hra-workflows-runner	A
HRA Multi-LOD Renderer (1.0.0)	https://github.com/cns-iu/hra-multi-lod	A
HRA AMAP	https://github.com/cns-iu/hra-amap	P
VCCF Visualizations	https://github.com/cns-iu/hra-vccf-cell-distance-visualizations	P
Vascular distance for 3D reconstruction of skin and spatial mapping of immune cell density	https://github.com/hubmapconsortium/vccf-visualization-2022	P
EBI ASCT+B Table Validation (2024-07-11)	https://github.com/hubmapconsortium/ccf-validation-tools	A
HRA 3D Reference Organ Validation	https://github.com/hubmapconsortium/hra-ref-organ-validation	A
**API for data access**		
HuBMAP Cells API	https://github.com/hubmapconsortium/cross_modality_query	H

HuBMAP Cells API (Python Client) (0.0.11)	https://github.com/hubmapconsortium/hubmap-api-py-client	H
HuBMAP Entity API (2.3.15)	https://github.com/hubmapconsortium/entity-api	H
HuBMAP Ingest API (2.3.17)	https://github.com/hubmapconsortium/ingest-api	H
HuBMAP Search API (3.3.12)	https://github.com/hubmapconsortium/search-api	H
API and SPARQL queries using the HRA-KG	https://github.com/hubmapconsortium/ccf-grlc	A
Ontology API for HuBMAP and SenNet applications (2.0.3) (extends UBKG API [2.1.4])	https://github.com/x-atlas-consortia/hs-ontology-api https://github.com/x-atlas-consortia/ubkg-api	H
HuBMAP UUID API (2.4.3)	https://github.com/x-atlas-consortia/uuid-api	H
HRA API (0.8.0)	https://github.com/x-atlas-consortia/hra-api	A
Tissue Block Annotation (1.0.0)	https://github.com/hubmapconsortium/hra-tissue-block-annotation	A
**User interfaces**		
HuBMAP Consortium	https://hubmapconsortium.org	H
HuBMAP Data Portal (0.102.5)	https://github.com/hubmapconsortium/portal-ui, https://portal.hubmapconsortium.org	H
HRA Portal	https://github.com/hubmapconsortium/hra-ui/tree/main/apps/humanatlas.io	A
Vitessce (3.4.6)	https://github.com/vitessce/vitessce	H
ASCT+B Reporter (2.8)	https://github.com/hubmapconsortium/hra-ui	A
EUI (3.8)	https://github.com/hubmapconsortium/hra-ui	H
RUI (3.8)	https://github.com/hubmapconsortium/hra-ui	H
FTU Explorer (0.5.0)	https://github.com/hubmapconsortium/hra-ui	P
HRA Organ Gallery (0.11.2)	https://github.com/cns-iu/hra-organ-gallery-in-vr	P
HRA Data Dashboard (0.1.0)	https://github.com/hubmapconsortium/hra-data-dashboard	P
VCCF Cell Distance Visualizations (0.1.0)	https://github.com/hubmapconsortium/hra-ui	P
Learn about the HRA through scrollytelling!	https://github.com/cns-iu/hra-scrollytelling	P
HRA Pilot Previews	https://github.com/hubmapconsortium/hra-previews	P
**Data / Ontologies**		
HuBMAP Ontology (CCF v1.x)	https://github.com/hubmapconsortium/hubmap-ontology	
Human Reference Atlas Knowledge Graph (HRA-KG)	https://github.com/hubmapconsortium/hra-kg	
HRA Vasculature CCF	https://github.com/hubmapconsortium/hra-vccf	P
HRA Vocabulary (2.5.10)	https://github.com/hubmapconsortium/hra-vocab	P
HRAlit (0.5)	https://github.com/cns-iu/hra-literature	P
